# Dust Contamination and Environmental Cofactor Effects on Flexible Sensors and Antennas: A Review

**DOI:** 10.3390/s26206407

**Published:** 2026-10-10

**Authors:** Hector Medina, Eli Kries

**Affiliations:** School of Engineering, Liberty University, Lynchburg, VA 24515, USA

**Keywords:** flexible sensors, flexible antennas, environmental degradation, humidity, particulate contamination, temperature variation, moisture ingress, electrochemical degradation, encapsulation, mitigation strategies

## Abstract

Flexible sensors and antennas are increasingly deployed in wearable health monitoring, environmental sensing, and RFID technology, yet their reliability under environmental exposure remains insufficiently addressed. This review synthesizes the literature on the environmental degradation of flexible sensors and antennas, with particulate-induced degradation as the primary stressor and humidity and temperature treated as cofactors; additional mechanisms such as flexural fatigue and UV-induced photodegradation are briefly addressed but beyond the scope. Across the literature, dust-induced degradation rarely acts independently: moisture ingress within the substrate repeatedly appears as a shared vulnerability that could: (a) shift dielectric properties, (b) enable moisture transport about the conductor surface, and (c) promote electrolyte formation through ionic particulate deposition, accelerating electrochemical degradation. Temperature further amplifies these mechanisms by accelerating moisture diffusion and reaction kinetics and by promoting the generation of microcracks through CTE mismatch and cyclic fatigue, which create sites for moisture ingress and particulate deposition. We conclude with a layered mitigation architecture spanning encapsulation, substrate selection, conductor and material engineering, and geometric design. Building on this architecture, we propose a provisional (prototype-like) three-axis design framework that classifies deployment environments by aqueous ionic conductivity of particulate deposits, sustained relative humidity, and operating temperature with cycling amplitude, mapping each of the 27 combinations to a candidate mitigation prescription. We identify key experimental gaps; most notably, the scarcity of dust deposition studies on flexible sensors and antennas and the absence of comparative substrate testing across the three stressors, simultaneously. Addressing these knowledge gaps could enable the development of mechanically robust, environmentally resilient flexible systems for wearable technologies, while also facilitating the exploitation of dust-induced surface degradation as a functional transduction mechanism for sensing applications. A Limitation Section is included to highlight the fact that sufficient particulate-deposition studies on flexible sensors and antennas remain elusive, which provides opportunities for future research. While some mechanistic effects and material characterization are extrapolated from rigid PCB and MEMS studies, this does not constitute direct experimental support for their flexible counterparts.

## 1. Introduction

Flexible sensors and antennas have emerged as an enabling technology in many applications, from wearable health monitors to environmental sensing and radio-frequency identification (RFID) in logistics and supply chain management [1,2,3,4,5,6,7]. The appeal of these devices lies in (a) their compliance, the ability to stretch and conform to curved, irregular, or moving surfaces that rigid electronics cannot conform to, (b) characteristics such as thinness, lightweight, and high flexibility that make them ideal for wearable applications, and (c) the scalability and cost advantages of non-contact fabrication methods such as inkjet printing, slot-die printing, or screen printing [8,9,10,11,12,13,14,15,16,17]. This combination has driven rapid growth in both research and commercial deployment, covering substrate classes from polyimide and PET films to textiles, paper, and nanocellulose, with conductor materials ranging from silver nanoparticle inks to carbon nanotubes and high-entropy nanofibers [18,19,20,21,22]. However, as these devices transition from controlled environments to real-world deployment, they face an environmental reliability problem that has not been properly characterized in the literature to date.

Among the environmental stressors that degrade flexible sensors and antennas, particulate contamination is uniquely consequential as its degradation mechanism is both chemically aggressive and modular to cofactors that are nearly always present in deployment. Particulate deposition of ionic and hygroscopic components forms electrolyte layers on the device surface, driving electrochemical corrosion and electrochemical migration (ECM) between adjacent conductors. However, this chain does not operate in isolation: humidity assists in dissolving the ionic content of the deposit and is accelerated by temperature, which increases the moisture diffusion rates and electrochemical kinetics. Humidity and temperature each produce independent, distinct degradation pathways—humidity disrupts substrate dielectric properties and enables moisture-induced oxidation, leakage currents, and delamination at the conductor–substrate connection; temperature introduces electrical drift, CTE mismatch deformation, and cyclic fatigue cracking—but these mechanisms are most consequential in flexible electronics when they act coincidentally with, and amplify, dust-induced degradation [23,24,25,26,27].

Treating each factor in isolation obscures the dependent nature of dust degradation in deployed environments. Humidity is not only a parallel stressor but also a necessary enabling condition: without sufficient relative humidity to form a surface electrolyte film, even the most ionic deposits remain electrochemically inert [23,26,28]. Temperature acts as an accelerating cofactor that increases moisture diffusion through polymer substrates and enhances the kinetics of electrochemical reactions induced by dust-induced electrolytes [23,26,27,28]. Furthermore, thermal cycling generates microcracks in substrate and conductor traces which accelerate moisture ingress and provides rigidity for particle entrapment, amplifying degradation chains that occur in entirely different domains. Evaluating these stressors independently consequently prevents a unified framework for predicting and designing flexible sensors and antennas that will perform under the variable environments which they will experience in deployment.

This review addresses this gap by synthesizing the existing literature on the effects of particulate contamination, humidity, and temperature on flexible electronics, with a crosscutting framework that emphasizes multistressor interactions with shared material properties. Evidence derived from rigid PCB or MEMS studies is utilized for material and mechanistic characterization but does not establish direct experimental support for flexible devices. Rather than addressing each stressor in isolation, the analysis identifies material properties as governing variables that mediate vulnerability across multiple environmental stressors.

**Figure 1:**
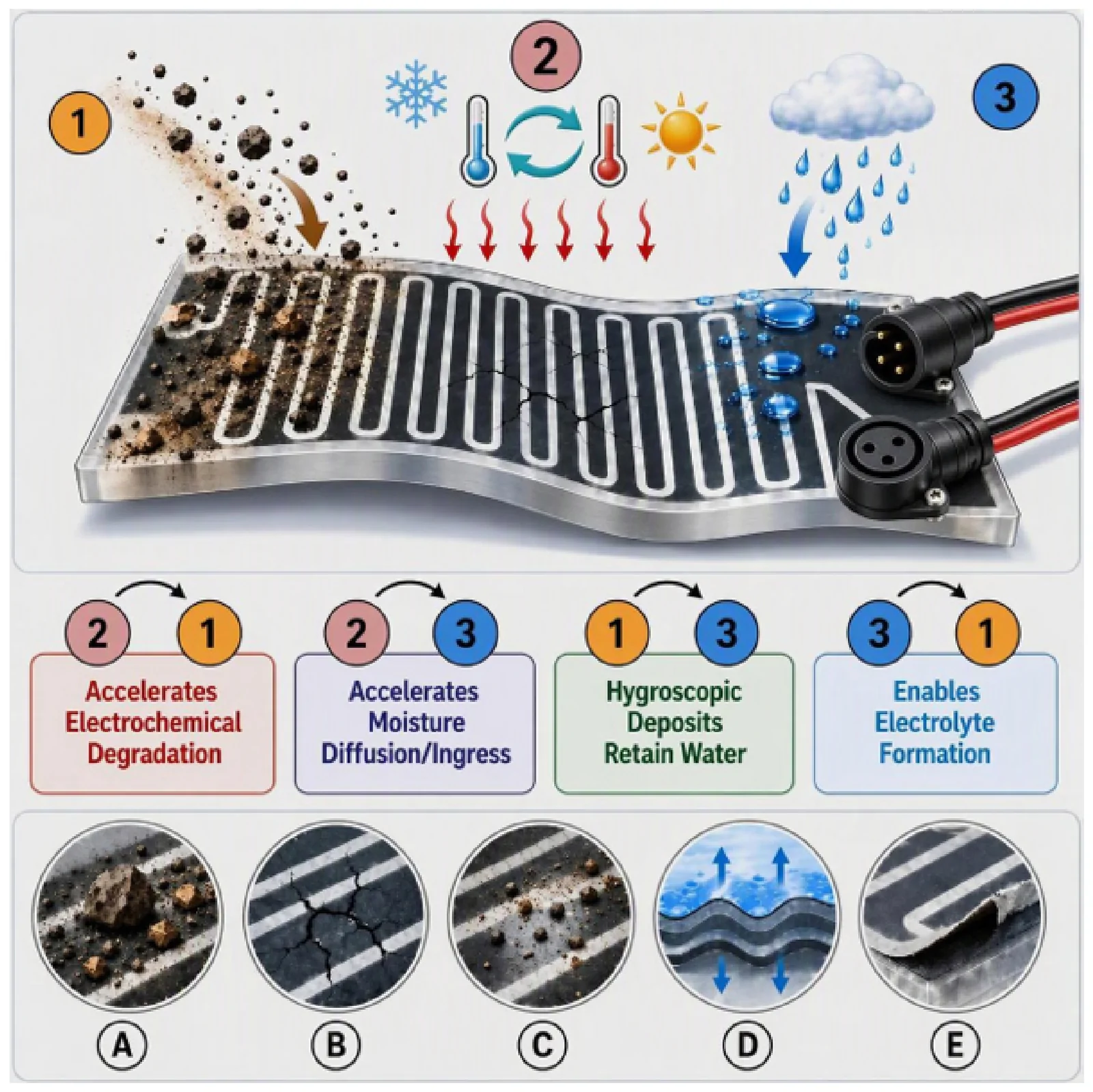
The effects of stressors such as (1) dust, (2) temperature, and (3) humidity on flexible sensors/antennas—or any other soft surface for that matter—must be taken into account in a combined fashion, since these are not independent mechanisms. For example, temperature (2) on dust (1) tends to accelerate electrochemical degradation; also, temperature (2) on humidity (3) tends to accelerate moisture diffusion; likewise, dust (1) on water/humidity (3) increases surface area for higher condensation probability; furthermore, humidity (3) on dust (1) enables the formation of electrolytes. These combined stressors lead to the acceleration of processes such as (A) abrasion, wear, or smearing, (B) crazing, mud-cracking, and surface cracks, (C) surface impurities and discoloration, (D) wrinkling, swelling, creasing, or softening, and (E) delamination, blistering, and interface shearing.

As shown in Figure 1, particulate contamination, humidity, and temperature are not independent mechanisms but a coupled system. Humidity serves as an enabling bridge for particulate-driven electrolyte formation, while temperature kinetically accelerates both moisture ingress and electrochemical degradation. This map visualizes a central claim of this review: environmental reliability in flexible electronics must be treated in tandem rather than as three isolated mechanisms. Although several existing reviews address flexible electronics broadly, to the authors’ knowledge, very few, if any, have addressed particulate contamination, humidity, and temperature-induced degradation mechanisms for flexible sensors and antennas together, identified the cross-interaction between them, and framed the material choice as a multistressor optimization problem. The principal contribution of this work is two-fold: (a) a synthesis that treats environmental reliability as a coupled system rather than isolated mechanisms, and (b) a prospective three-axis framework (Section 4.2) that translates this synthesis into environment-specific mitigation prescriptions, providing a structured basis for material and geometric selection that the existing literature has yet to offer.

## 2. Scope and Methodology

This review restricts its scope to particulate (dust) contamination as the primary degradation stressor, with humidity and temperature treated as cofactors; thus, the existing literature on flexible sensors and antennas provides sufficient quantitative evidence to support the degradation mechanisms and classifications derived. Additional factors, such as ultraviolet radiation and mechanical deformation, are briefly discussed in Section 5, but are not incorporated into the framework developed in Section 4. This review focuses primarily on flexible sensors and antennas fabricated on flexible polymer substrates (polyimide, polyethylene terephthalate, fluorinated ethylene propylene, paper, and textiles) with printed or deposited conductors (silver-, copper-, gold-, and carbon-based inks). Rigid substrates are excluded except where their material and mechanistic properties can be contextually transferred to flexible substrates, as expressed in Section 3.1.

The literature was identified through structured keyword searches via Google Scholar, supplemented by key reference citation tracking. The terms of *the* “*flexible antenna*” “*dust contamination*,” “*electrochemical migration (ECM)*,” “*ionic conductivity*,” “*humidity induced degradation*,” “*moisture/humidity ingress*,” “*thermal cycling*,” *CTE mismatch* and mitigation mechanism: “*encapsulation*,” “*substrate selection*,” “*conductor material*,” and “*fractal geometry*”. Additional keywords for the targeted sources included “*dendritic growth*,” “*critical relative humidity*,” “*CTE in polymers*,” “*ionic content of common dusts*,” and “*PCB dust contamination*, as review gaps were identified. No date restriction was used; the selected resulting studies span from electrochemical migration studies of the 1980s to recent publications (2025–2026). Approximately 300–400 publications were selected by title and abstract, of which 112 were selected for inclusion.

Publications were included if they (a) reported experimental or simulated evidence of degradation on flexible substrates or printed circuit boards under particulate contamination, humidity, or temperature; (b) characterized material properties (moisture ingress, CTE, TCR) or (c) baseline values (conductivity of aqueous dust and critical relative humidity); or (d) demonstrated mitigation strategies with quantified performance data. Publications were excluded if they utilized rigid electronics without a relevant material or mechanistic context, addressed fabrication processes without relevant reliable characterizations, or if the described mechanisms fell entirely outside the scope of the review. While this review follows a narrative synthesis approach, Table 1 summarizes the selected studies by device construction, contamination and exposure protocols, performance metrics, mitigation approaches, major findings, and transfer limitations. The design considerations, environmental classification thresholds, and prospective design classification developed in Section 4 were the result of the synthesis of the reviewed literature rather than the analytical methodology.

## 3. Degradation Mechanisms

### 3.1. Dust and Particulate Contamination

Dust is not simply a single uniform substance, but a heterogeneous mixture of inorganic minerals, organic fibers, ionic salts, and hygroscopic compounds whose composition varies distinctively between environments [23,26,27,50,51,61]. Indoor environments concentrate sources that are disproportionally salt rich. Human skin and sweat carry NaCl and KCl, cleaning aerosols contain sulfate and phosphate, and building materials release calcium sulfate and carbonate. In a confined space, the ionic composition of the indoor dust is much greater than that of the mineral-rich outdoor dust. Thus, indoor dust exhibits a mean aqueous conductivity of 2526 μS/cm, markedly higher than 1640 μS/cm of outdoor dust [23,26]. This distinction means that the severity of dust-induced degradation in an electronic device may depend not only on the mass of the particulate but also on its pH, hygroscopic composition, and ionic salt content [23,26,27,50,51,61]. It should be noted that the literature surrounding dust-induced degradation in flexible electronics is scarce; therefore, much of the evidence reviewed here is extrapolated from PCB and MEMS research. Evidence from rigid PCB and MEMS supports electronic mechanisms and properties, but does not constitute direct experimental support for flexible devices. The electrochemical processes of ionization, corrosion, and ECM demonstrated by Song et al. are governed solely by electrolyte chemistry and circuit board geometry, not by substrate rigidity [23,26]. The theoretical basis for transferability to flexible substrates is developed throughout this section.

The degradation of electronics via dust contamination is not an independent phenomenon but forms a linked electrochemical chain. First, particulate deposition on an exposed surface’s hygroscopic contents absorbs ambient moisture, forming a thin aqueous film atop the surface once the relative humidity (RH) exceeds the critical relative humidity (CRH) specific to a given salt. Song et al. identified CRH values ranging from 40% for ammonium bisulfate to 79% for ammonium sulfate at room temperature, such that typical indoor humidity conditions (40–60% RH) are sufficient to activate the electrochemical chain [26]. This film then dissolves the ionic salts present in the dust, creating an electrolyte between the adjacent conductors [23,26,27]. Once this electrolyte is introduced, two degradation mechanics are carried out—electrochemical corrosion and electrochemical migration (ECM)—where metal ions dissolve and migrate from the anode through the electrolyte (with an applied current) and deposit as dendritic growths about the cathode [23,26,27,48,62]. These dendrites can bridge conductor gaps and can cause short circuiting or failure. Minzari et al. further characterized the macro-, micro- and nanoscale morphology of tin dendrites formed under ECM, providing structural evidence and characteristics for the formation of dendrites under ECM [62]. The critical insight from Song’s thesis is that each step in this degradation is varied by dust content [23]. Dust with a higher ionic content and a lower pH accelerates electrolyte formation, whereas more inert particulate may cause comparatively less damage.

Among the limited studies addressing ECM on flexible substrates directly, Noh et al. evaluated electrochemical migration on flexible PCBs with copper, electroless nickel, and electroless nickel-immersion gold surface finishes [29]. Using water-drop testing, they found that the susceptibility of ECM in flexible PCBs exhibited the same characteristics and trends observed in rigid boards: the dendrite growth rate decreased in the order Cu > electroless Ni > NiAu, and migration accelerated with decreasing electrode spacing and increased voltage [63]. Critically, the ECM mechanism itself was not altered by the polyimide substrate, suggesting that the electrochemical degradation chain established on rigid PCBs may be mechanistically transferrable to flexible devices. However, note that Noh et al. used clean water droplets instead of ionic electrolytes. Zhou et al. further corroborated and extended this characterization by testing ECM on a silver-immersion PCB [48] Depositing both natural dust solutions and representative ionic salts such as NaCl and Na_2_SO_4_ degraded the PCB through loss of surface insulation resistance and dendritic bridging. Their results demonstrated that the ionic salt present in the particulate produced distinct failure characteristics and ECM, demonstrating that the severity of degradation is environmentally dependent [48,51,61,63,64]. Similarly, Lu et al.’s recent ECM study deposited solutions of NaCl and Na_2_SO_4_, to simulate common ionic salts in nature, in an Sn-58Bi solder to analyze degradation mechanisms via pollutants [49] They reported mean failure times of 53 and 32 s for NaCl and Na_2_SO_4_ solutions, respectively. The speed of these failures, combined with the variation in the failure time between salts, illustrates how rapidly ECM degradation can occur in the presence of an electrolyte and the characteristic that ECM degradation may be governed by the particulate’s content [49,50,51,61,63,65]. Collectively, Zhou et al. and Lu et al. demonstrate that the ionic composition of dust governs both the speed and mechanisms of ECM.

Jellesen et al. documented deployed device corrosion failure in commercial electronic devices caused by residues, providing field evidence that laboratory-characterized mechanisms mentioned above also manifest in deployed systems. Their findings further demonstrate that the severity of the corrosion within the device was governed by the content of the particles [50,51].

**Figure 2:**
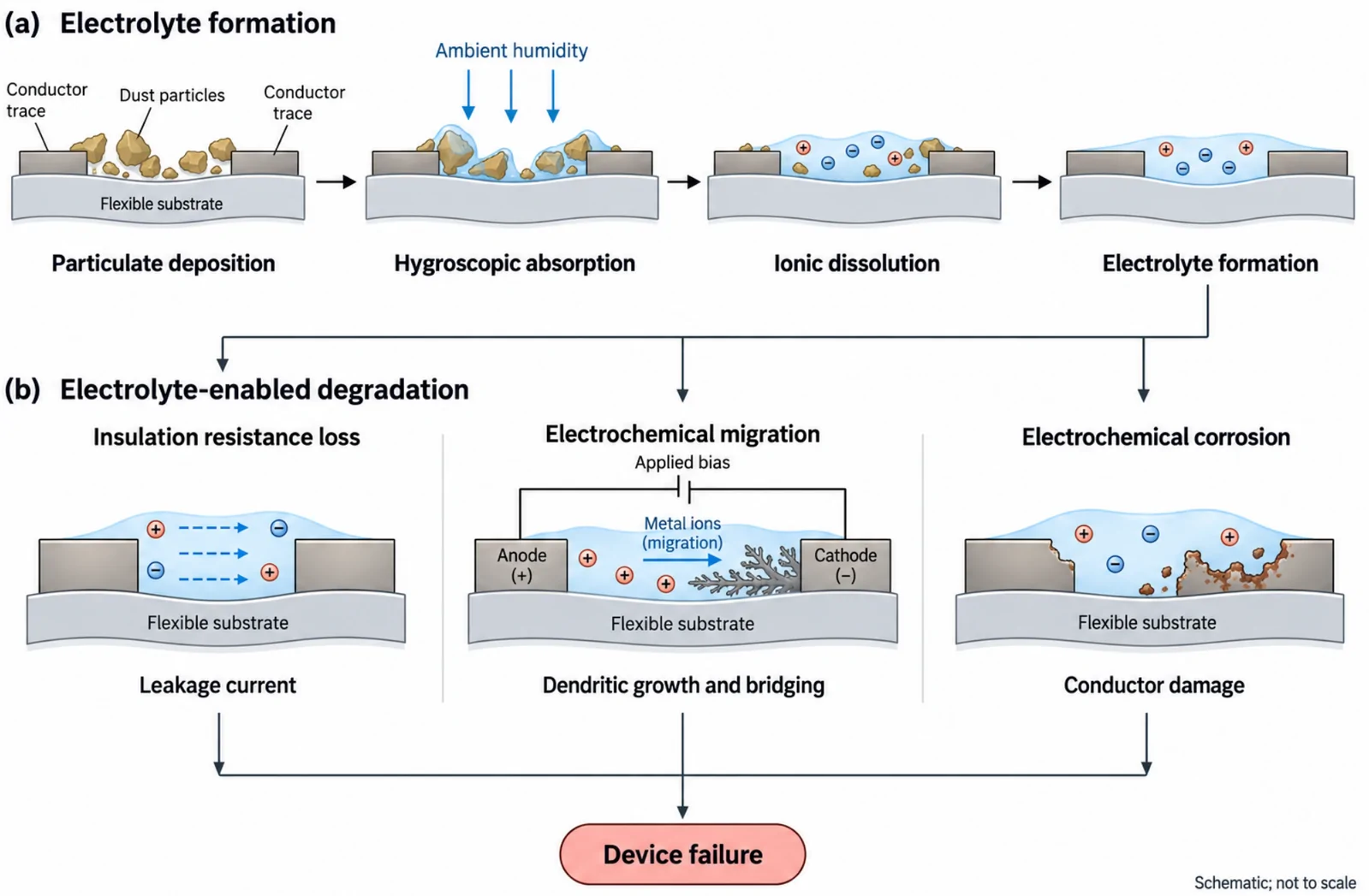
Moisture-enabled particulate pathways. A liquid film may permit leakage and corrosion and, with suitable electrical bias and electrode spacing, electrochemical migration and dendritic growth. These are possible pathways, not inevitable progression to device failure.

As demonstrated in Figure 2, the electrochemical chain illustrated above is enabled in the presence of ambient humidity. Humidity activates the formation of the electrolyte layer that enables corrosion, electrochemical migration, and resistance loss, which may ultimately lead to device failure. In alignment with a central argument of this review, particulate contamination must be evaluated with the hygrothermal environment, not in isolation.

Alongside the electrochemical chain, dust introduces a separate but parallel degradation mechanism through electrostatic currents (ESCs). Chen et al. combined a MATLAB R2022b-based simulation with dust-exposure tests, demonstrating that particulates generate measurable electrostatic currents through triboelectric friction during deposition. These ESCs interfere with the intended signal from the sensor, introducing noise that degrades the accuracy of the sensor [53]. They further demonstrated that electrostatic interference was attenuated under elevated humidity [53]. They established electrostatic disturbance as a distinct pathway through which dust may compromise *sensor* performance independently of the electrochemical corrosion described in [23].

Quantitative evidence from microelectromechanical systems (MEMSs) corroborates the effects of dust-induced degradation, though evidence is not intrinsically transferrable. Yi et al. subjected a MEMS thermal wind sensor to prolonged dust loading and measured a 3.75% reduction in voltage output at a dust density of 0.12 g/mL. The reduction in output was scaled proportionally with the concentration of particles [52]. Similarly, in a PurpleAir™ particulate sensor deployment study, de Souza et al. found that ≈2% of the units met their criterion for “permanent degradation”, with losses increasing at a rate of 0.93% annually [54]. Yi et al. suggest that prolonged accumulation of particulate can progressively alter the accuracy of the sensor. However, they represent implications on rigid substrates with relatively smooth surfaces; flexible substrates are not mechanistically equivalent.

#### 3.1.1. Synergistic Effects

In each of these studies, the interaction between dust, temperature, and humidity demonstrates a synergistic relationship rather than an additive effect [26,27]. Song’s thesis demonstrated that the electrochemical degradation mechanisms are fundamentally humidity-dependent. Without sufficient relative humidity (or CRH) to form a surface electrolyte, ionic particulates can remain inert [26]. Moreover, the finding that indoor dust exhibits substantially higher conductivity than outdoor dust (2526 ≫ 1640 μS/cm) suggests that *enclosed environments* with sustained humidity levels may pose greater contamination risks than settings with lower sustained humidity [23,26,27]. Chen et al. reinforces this synergistic principle through electrostatic currents, showing that relative humidity can impact the magnitude of triboelectric charges in the resultant interference [53]. Piotrowska et al. further demonstrated that, in the solder-flux activators studied, increasing temperature alters the critical relative humidity for water-vapor absorption to lower values in contaminated sources [55]. This implies that contaminated devices can form conductive electrolytes in less humid environments than clean ones. Together with temperature, this effectively reduces the environmental threshold at which the electrochemical degradation chain is activated, highlighting the synergistic properties between temperature, contaminants, and humidity. The synergistic effect of humidity and temperature on dust-related degradation mechanisms implies that dust contamination cannot be assessed in isolation from the hygrothermal environment, whose consideration directly intersects with the humidity- and temperature-related synergistic degradation mechanisms reviewed in Section 3.2 and Section 3.3.

#### 3.1.2. Mitigation Strategies

The preliminary review suggests that physical barriers can partially mitigate the effects of dust and humidity contamination on sensor performance. Chen, L. et al. demonstrated that incorporation of a polytetrafluoroethylene (PTFE) filter over a MEMS gas sensor reduced moisture-related degradation by a mean of 60.6% at 75% RH relative to an unfiltered package [56]. Although this study mainly addressed humidity filtration rather than minimizing particulate contamination, the PTFE filter may inherently serve as both a humidity barrier and a particulate barrier, as expressed in Section 3.1.1. Though, the study did not measure particulate-filtration performance or test this approach on flexible devices. Also, among the literature on ECM in flexible substrates directly, Noh et al.’s evaluation of ECM on flexible PCBs demonstrated that increasing inter-electrode spacing reduces the likelihood and speed of electrochemical dendritic bridging by extending the migration path length and delaying dendritic closure under particulate-induced degradation [29]. More mitigation strategies for flexible devices are presented in Section 4.

#### 3.1.3. Limitations and Caveats

Despite the well-established understanding of dust-induced degradation mechanisms in rigid electronics and MEMS research, a critical gap remains: direct studies on dust deposition degradation mechanisms on flexible electronics appear sparse; most evidence is extrapolated from rigid PCBs/MEMS. Although Song’s [23] electrochemical characterization, Yi et al.’s [52] MEMS sensor experiments, Chen et al.’s [53] simulation and MEMS tests, and de Souza et al.’s [54] field analysis provide foundations and quantitative benchmarks, few, if any, studies have tested dust-induced degradation of flexible substrates specifically; the ECM mechanism has been confirmed on flexible PCBs by Noh et al. [29], but experimental procedures used clean water, not dust-derived electrolytes. Also, industry standards such as MIL-STD-2000 define water-soluble contamination limits for rigid soldered electric and electronic assembles, yet, to the authors’ knowledge, equivalent cleanliness thresholds have seldom been established for flexible electronics [66]. Given the rapid proliferation of flexible electronics, the experimental validation of dust degradation on flexible sensors and antennas represents an unaddressed research field, further discussed in Section Research Gaps and Validation Priorities.

### 3.2. Humidity: Enabling Cofactor and Independent Mechanisms

Humidity exists as an ongoing and pervasive environmental challenge for flexible sensors and antennas; one that is intrinsic to nearly every deployment context that flexible electronics are designed to serve. Wearable electronics often operate in the sustained presence of body perspiration and moisture; health monitoring devices operate under constant phase changes in humidity; and outdoor sensors face fluctuations in diurnal and seasonal humidity. Unlike most rigid electronics, which may be enclosed under controlled internal atmospheres, flexible devices can be constrained by their requirement for compliance. Similarly, often sought after for its application in wearable technology and environmental measurements, enclosure is often not an option. Consequently, the resilience of flexible electronics to humidity is governed primarily by the substrate material itself, particularly its capacity to absorb, retain, and transmit moisture. Substrate-level moisture uptake, which includes both capillary wicking through porous fibrous networks and hygroscopic absorption into polymer chains, emerges as a key variable to the degradation mechanisms of humidity across substrates; and, as noted in Section 3.1, can synergistically govern susceptibility to dust-induced electrochemical degradation.

The first mechanism through which humidity degrades the performance of flexible devices involves moisture uptake through the substrate to reach the electronic components. In fibrous substrates such as textiles and paper, this uptake can occur through capillary wicking along its interconnected networks. Polymer films can also absorb water, with material-dependent effects on their electrical properties, as discussed below [21]. Watanabe et al. quantified the equilibrium ratio of moisture absorption for common flexible substrates after immersion, where PMDA-ODA, BPDA-PDA and PET were 2.7, 2.5 and 0.5%, respectively [57]. Importantly, the resistivity of both polyimides decreased sharply once water exceeded approximately 3.4% by volume, at which point moisture within the film formed conductive pathways throughout the film. Similarly, Xiao et al. highlighted nanocellulose as a particularly moisture-sensitive substrate, consistent with the hygroscopic vulnerability of paper-based devices [20,21,22].

Moisture diffusion can be caused by a substrate’s (often polar) affinity for water. Lu et al. provided a comprehensive review of moisture transport and interaction mechanisms across various flexible humidity sensors [67]. Once at the interface, moisture initiates a series of degradation mechanisms: oxidation of metallic traces increases resistance; currents leak over adjacent conductors through the moisture; and adhesion between the functional layer and the substrate weakens [68]. Bollström et al. demonstrated this pathway on inkjet-printed electrodes on a paper substrate. Exposed to 90% RH for 1 h, 18 h in a dry glove box, and another hour at 90% RH, the transistor on/off current ratio ranges from 22 to 7 [30]. Their study demonstrated that a multilayer barrier that covers the paper substrate improved dimensional stability by reducing moisture penetration, not only offering a potential mitigation strategy but directly confirming that the rate of moisture migration through a substrate can control the severity of the degradation [30]. At the conductor interface, Andersson et al. demonstrated that inkjet-printed silver nanoparticles on a paper substrate exhibit a non-reversible decrease in resistance when exposed past a critical humidity threshold [31,69]. Prolonged exposure to various levels of relative humidity (RH) revealed that a variation in resistance values reflects the impact that the geometry of the conductor has on the resistance values under external stressors. Unlike substrate dielectric variation, this conductor/ink-specific degradation mechanism indicates that moisture interacts with the conductor structure itself. This vulnerability extends beyond silver-to-copper-based inks. Tomotoshi et al. documented that the resistivity of copper ink increased from 172 μΩ·cm to 1.02 Ω·cm after 24 h at 85 °C and 85% RH [70,71]. However, it should be mentioned that paper substrates degrade through mechanical contractions and swelling mechanisms unique to cellulose networks and vary drastically from the moisture transportation processes in polymer-based films. However, the underlying principle of substrate permeability determines the magnitude of conductor degradation regardless of the substrate class. In any case, the oxidation, cracking, and delamination produced by moisture penetration cause irreversible structural damage, permanently compromising the device.

The second mechanism through which humidity may degrade the performance of flexible devices is dielectric disruption within the substrate. When a substrate absorbs ambient moisture, the water molecules occupy interstitial spaces within a paper-based substrate, porous fiber networks, or in a polymer substrate, into the polymer chain structure of dense films, increasing the dielectric constant of the substrate [72,73]. Hertleer et al. demonstrated this effect quantitatively on textile patch antennas, reporting that both the relative permittivity and the loss tangent increased with increasing relative humidity on all five tested substrates. The magnitude of this dielectric shift was governed by the moisture regain of the substrate. Cotton, with a moisture regain of 7.33%, exhibited a large increase in permittivity that shifted antenna resonance frequency downward, while polyester, regaining only 0.95%, showed a minuscule deviation under identical conditions [25]. Pérez-Campos et al. adds to these findings with dielectric measurements of textile substrates at 2.45 GHz, confirming that significant permittivity shifts may occur under variations in temperature, density and humidity [58]. El Gharbi et al. corroborated these dielectric variations in a broader range of textiles, reporting dielectric constants ranging from 1.215 for felt to 1.9 for PET, with loss tangents in the range of 0.0045 for polyester and 0.05 for denim [1]. Note that, unlike the first mechanism, this pathway can be reversible: antenna performance may recover as the substrate dries.

**Figure 3:**
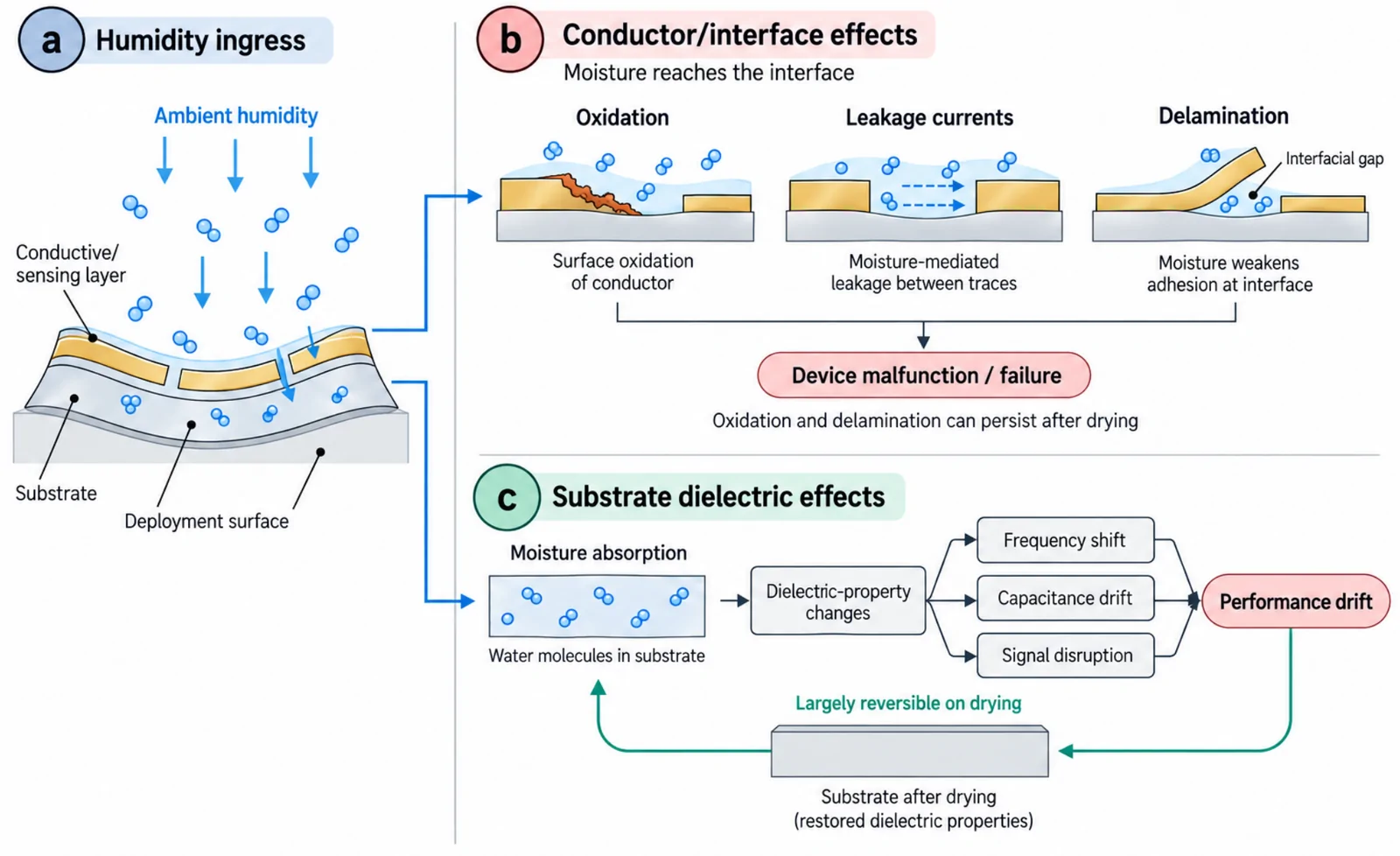
Dual-pathway degradation mechanisms induced via humidity ingress: (path a to b) moisture transport to the conductor interface, and (path a to c) moisture-induced change in substrate dielectric properties. Conductor interface effects do not universally lead to device failure, nor are dielectric effects universally reversible.

Figure 3 summarizes the two distinct pathways through which humidity degrades flexible devices. The upper pathway demonstrates moisture penetration to the device’s conductor interface, which may lead to oxidation, leakage currents, and delamination, which ultimately leads to irreversible damage and device failure. The lower pathway represents dielectric disruption via the device’s substrate, which can cause reversible effects such as frequency and capacitance drift and signal disruption. Humidity exposure does not produce a single path of failure, but a split path dependent on the material through which moisture is present.

The relationship between substrate moisture regain is not always straightforward. Under prolonged humidity exposure, the results of common flexible substrate material diverge and vary significantly. Lahokallio et al. subjected polyimide, polyethylene terephthalate, and fluorinated ethylene propylene films to accelerated aging at 85 °C and 85% RH for up to 6000 h, thus evaluating the change in elasticity, glass transition temperature, melting point, thermal expansion, moisture absorption, and crystallinity [28,71,74]. Zardetto et al. corroborate plastic insulating films, polyethylene naphthalate (PEN), as a substrate alternative comparable to PET and PI, with intermediately characterized moisture resistance and thermal stability [32]. PET, which absorbs ≈0.5% water by mass, hydrolyzes its bonds, causing progressive embrittlement, rendering it too embrittled after 2000 h. FEP absorbed negligible moisture (≈0.05%) and maintained a more stable structure; however, it progressively exhibited increased crystallinity and signs of oxidation as the experiment continued. PI absorbed the highest proportion of water among the three materials tested at 2% water by mass. Yet, paradoxically, PI remained the most stable throughout the 6000 h [28]. This contradiction reveals an inherent caveat of the application of this study: substrate survival does not equate to device survival. Despite PI resisting moisture-induced degradation, its high absorption demonstrates that, without an inter-substrate barrier, it may sustain water availability to the interface. As such, this interpretation aligns with findings by Frisk et al., who reported that flexible PCBs with polyimide substrate exhibited lower reliability in humidity testing than liquid crystal polymer alternatives [33]. For future flexible device designs, this implies that the selection of the substrate must consider not only the humidity tolerance of the materials but also its role as a moisture transport medium for functional circuitry.

#### 3.2.1. Synergistic Effects

Humidity-induced degradation does not operate in isolation from other environmental stressors, but it can be synergistically accelerated by temperature and by particulate contamination. The relationship between temperature and moisture degradation is largely governed by fundamental physical chemistry: elevated temperatures increase the kinetic energy of water molecules, which increases both their diffusion rate through polymer matrices and the reaction rate of hydrolysis at susceptible molecular bonds. This relationship follows Arrhenius kinetics, meaning that even modest temperature increases produce an exponential acceleration of degradation. Bollström et al. noted that thermal effects amplified the moisture-induced degradation of their PCBs [30]. At the assembly level, Davila-Frias et al. investigated temperature–humidity degradation in flexible hybrid electronics comprising a thinned silicon die bonded to traces on a flexible substrate. Five samples per condition were tested for 150 h at four combinations of 65 or 85 °C and 85 or 98% RH, with the changes in resistance recorded every five hours [34]. Using a 20% increase in initial resistance as the failure criterion, the authors estimated mean pseudo-failure times ranging from 143.8 h at 85 °C/98% RH to 913.1 h at 65 °C/85% RH [34]. The study therefore provides experimental resistance-degradation data for the tested assembly, not direct, validated measurements. Gupta et al. separately varied relative humidity and temperature while loading a screen-printed stretchable strain-sensing array, finding changes in its resistance response and baseline while the strain response remained approximately linear [36].

In wearable applications, perspiration introduces a unique form of humidity exposure that not only compounds the degradation mechanisms mentioned above but works synergistically with the electrolytic degradation described in Section 3.1. Sweat is not only pure water but an ionic solution containing sodium chloride, potassium, lactate, and other electrolytes at significant concentrations. Sandeep et al. quantified this effect by exposing a textile antenna to normal humidity and simulated sweat conditions (a standardized solution comprising 19 amino acids, seven minerals, and four metabolites (including uric acid, lactic acid, and urea) at pH 4.5), measuring a 718-fold increase in the corrosion rate under sweat exposure compared to “normal conditions”. Full immersion resulted in a 79.58% increase in substrate weight, indicating substantial absorption into the textile [37]. The significance of these findings extends beyond the immediate data: sweat can collapse the distinction between humidity degradation and the dust-induced electrochemical degradation chain expressed in Section 3.1. However, where dust degradation requires ambient humidity to dissolve ionic particulate deposits, sweat delivers a pre-dissolved electrolytic solution directly onto the substrate surface [23,26]. In a worn device, electrochemical corrosion, ionic contamination, and electrochemical migration can proceed without any dust contamination whatsoever. Sweat may be sufficient to activate the full electrochemical degradation chain. As established in Section 3.1, the electrochemical degradation chain initiated by the particulate is humidity-dependent, requiring a surface moisture film to form the electrolyte that progresses the degradation [26,27]. This implies that humidity is not a far-removed degradation stressor but may be necessary to allow dust-induced degradation. The moisture absorption of the substrate governs the way in which the electrolyte layer forms readily and persistently in the presence of dust contamination. Thus, substrate moisture management is a critical design parameter with implications for multiple environmental stressors.

#### 3.2.2. Mitigation Strategies

Inter-substrate barriers and encapsulation strategies offer partial mitigation of humidity degradation in flexible devices [75]. Zou et al. demonstrated that Ecoflex encapsulation of a flexible hydrogel sensor reduced the change in humidity weight by 94.2% and the change in resistance from 580.8% to 7.7% over one month of exposure at 60% RH, suggesting that barrier coatings in polymer substrates can substantially inhibit moisture absorption without sacrificing flexibility [38,75]. Similarly, the multilayer barrier coating demonstrated in Bollström et al. [30] similarly reduced moisture penetration on paper substrates by improving dimensional stability. The findings of Hertleer et al. suggest that material selection itself constitutes a mitigation strategy by design, where specifying substrates with low moisture regain inherently reduce dielectric vulnerability before any protective barrier is applied [25]. Chen et al. further demonstrated that incorporating a PTFE filter into the MEMS gas sensor packaging reduced the sensor’s humidity-induced response by 60.6% relative to the packaging without the filter [56]. A comprehensive evaluation of these and additional mitigation strategies against humidity degradation in flexible devices is discussed in Section 4.

#### 3.2.3. Limitations and Caveats

The current existing literature on humidity degradation in flexible electronics, although more saturated and mature than the dust contamination research reviewed in Section 3.1, remains fragmented between substrate classes. Hertleer et al. [25] characterized dielectric disruption in textile antennas; Bollström et al. [30] documented damage on paper electronics; and Lahokallio et al. [28] evaluated polymer film degradation at the material level, but each study addressed only a single degradation mechanism in isolation, and none examined both the dielectric and penetration to conductor pathways within the same experimental design. To date, no study has systematically evaluated how substrate moisture absorption predicts the *relative* magnitude of humidity degradation across multiple substrate classes in flexible electronics. Moreover, the role of substrate moisture uptake in both humidity- and dust-induced degradation (Section 3.1) remains insufficiently investigated experimentally. Establishing this unified degradation mechanism through controlled comparative testing across substrate types and stressor combinations represents an unaddressed research opportunity in the field of flexible electronics, discussed further in Section Research Gaps and Validation Priorities.

### 3.3. Temperature: Accelerating Cofactor and Independent Mechanisms

Temperature variation remains a fundamental environmental stressor for flexible and rigid electronics alike, one that differs from humidity and dust contamination in a critical respect: thermal degradation is governed by the material properties of both the substrate and conductor and the interaction between them. Applications of flexible devices exist across thermal environments ranging from cryogenic aerospace applications to body-worn systems subject to high temperatures. Conventional polymer substrates, notably polyimide, PET, and polyvinylidene fluoride (PVDF), have material-specific thermal limits under sustained or cyclic thermal loading [24,76]. Li et al. identified thermal instability as a fundamental limitation of thermally degradable flexible electronic systems, noting that the temperature sensitivity of polymer substrates inherently constrains the operating applications of flexible devices [39]. The degradation mechanisms caused by thermal exposure can occur through three distinct pathways: electrical drift, mechanical deformation, and structural failure. Each is governed by unique material properties and each has different implications for mitigating thermal degradation.

One immediate degradation mechanism on flexible sensors is electrical drift: temperature-dependent changes in the electrical properties of conductor materials that alter the output. This mechanism arises fundamentally from electron–phonon coupling—as temperature increases, lattice vibrations intensify and scatter electrons more frequently, increasing the electrical resistivity of conductors [19,77]. The magnitude of this effect is quantified by the temperature coefficient of resistance (TCR), which varies between the various materials considered in flexible electronics. Liu et al. compiled TCR values for flexible thermistor materials. For metallic films and nanowires, where electron–phonon scattering is dominant, reported values cluster between about 0.002 °C^−1^ for silver and platinum films and 0.003 °C^−1^ for silver nanowires [78]. Considerably higher TCR values have been reported, including 0.5 °C^−1^ for silver nanocrystal films and 1.4 °C^−1^ for nanocracked gold films, using sensing mechanisms such as thermal expansion-driven cracking rather than intrinsic electron–phonon scattering [78]. For devices measuring non-thermal parameters, such as strain or electromagnetic sensors, this temperature dependence introduces signal crosstalk between the thermal and intended response of the sensing material, faltering accuracy [19,40]. Bhattacharjee et al. demonstrated temperature-dependent electrical response in a printed flexible antenna on a PVC substrate, measuring a 70% decrease in resistance as the temperature increased from 25 to 90 °C, while the measured *S*_11_ magnitude changed by approximately 3.5 dB [41]. Although electrical drift can be a reversible process. Once the temperature normalizes, the performance of the device returns to baseline (assuming that no structural damage has occurred). Nevertheless, in applications where constant measurement is needed under varying temperatures, electrical drift can render readings unreliable [2].

Furthermore, temperature variation induces mechanical deformation in flexible devices through differential expansion between the substrate and conductive layers. The coefficient of thermal expansion (CTE) of common flexible substrates may differ substantially from that of the conductive metals. PET exhibits a CTE of approximately 20–30 ppm/°C [79], while aluminum wiring has a CTE of ≈24 ppm/°C, and silicon, often used to attach the antennas, has a CTE below 3 ppm/°C [24,74,76]. When a multilayer flexible assembly is subjected to variations in temperature, these differing expansion rates can generate internal shear stresses at the material connections. Liu et al. investigated this mechanism on a flexible film deposited on a polyimide substrate, finding that thermal *cycling* induced internal stress that distorted the sensor geometry and degraded accuracy [47]. Their study further demonstrated that the distribution of thermally induced stress was dependent on the layout of the conductor itself: a conventional linear trace pattern fell victim to stress at bending points, whereas a Peano fractal geometry distributed stress more uniformly, reducing peak stress to 69.03% of its linear trace baseline [47]. The fractal conductor approach demonstrated by Liu et al. builds on the foundational work of Fan et al., whose conductor geometry experiments in stretchable electronics showed that deterministic fractal motifs enable space-filling electronic substrates with exceptional mechanical resistance [14,47]. These findings suggest that mechanical deformation under thermal expansion and contraction is not only a material issue, but a design problem: the conductor geometry translates the CTE mismatch into localized regions and can cause failures.

When thermal cycling is repeated, the cumulative mechanical and fatigue of repeated expansion and contraction can cause irreversible structural failure. Lahokallio et al. provided the most systematic investigation of this phenomenon, subjecting UHF RFID tags on PET to thermal cycling between −40 and 125 °C across four different profiles. The predominant degradation mechanism was the cracking of aluminum wire about the RFID circuit, notably the region of highest CTE mismatch between the PET substrate (70 ppm/°C), aluminum conductor (24 ppm/°C), and silicon chip (3 ppm/°C) [24,74]. Particularly, they found that the failure mechanism was sensitive to the cyclic profile: fast transition times with short soak periods produced the most accelerated antenna cracking, while longer soak times and extreme temperatures alternatively degraded the conductive adhesives between the chip and antenna [24]. Merilampi et al. corroborate these findings (on the RFID tags on paper substrate), observing that thermal cycling with high humidity can cause cracking in both the wiring and substrate connections, causing increased threshold power, decreased backscattered power, and shortened read ranges [35]. Accompanying this structural degradation, the shift in optimal operating frequency is particularly consequential for commercial RFID systems, relying on a narrow bandwidth and not being able to tolerate substantial frequency drift. It should be noted that both studies employed accelerated life testing with temperature extremes and cycling rates that exceed typical field conditions [80], and both authors cautioned that few accelerated thermal tests of RFID systems have been published. Thus, the relationship between accelerated test results and deployed reliability remains poorly corroborated.

It must be emphasized that the relationship between temperature and device performance is not exclusively detrimental. Conversely, Merilampi et al. reported that sustained exposure to elevated temperature, devoid of cycling or humidity, actually improved the conductivity of printed circuitry on their RFID devices, attributing this effect to thermally induced annealing that improved boundary connectivity and reduced sheet resistance in nanoparticle-based inks [35]. This behavior, where prolonged exposure to moderate heat can improve electrical performance, whereas cyclic temperature variation degrades it, complicates the assessment of thermal degradation for flexible devices. A device that performs well under prolonged heat as a result of the annealing effects may be overwhelmed by the mechanical effects of cyclic temperature and subsequent expansion and contraction. However, the improvement in conductivity from thermal annealing has primarily been observed in the absence of humidity, where even a prolonged high temperature combined with elevated humidity mitigated the annealing effect, demonstrating the synergistic coupling between thermal- and humidity-induced degradation mechanisms, discussed in Section 3.2 [35]. In any case, the design choices of the substrate and conductive material should be considered in conjunction with the environment in which it is intended to be used.

#### 3.3.1. Synergistic Effects

Thermal degradation does not operate independently of the dust and humidity contamination reviewed in Section 3.1 and Section 3.2, but interacts synergistically with both. Elevated temperature accelerates moisture diffusion about polymer substrates, increases the rate of hydrolytic degradation in moisture-prone materials such as PET, and enhances the kinetics of electrochemical reactions activated by dust-induced electrolytic degradation [23,28,55]. Merilampi et al. illustrated this coupling, finding that high temperature can paradoxically improve device performance via annealing, but in combination with high humidity, it can produce device degradation [35]. This improvement in conductivity under sustained heat is consistent with the documented behavior of metallic nanoparticle inks, where thermal energy controls particle boundaries and reduces grain boundary resistance [15]. Thermal cycling is another coupling mechanism, where repeated mechanical stress from CTE mismatch can generate microcracks in conductor and substrate materials alike, subsequently serving as pathways for moisture absorption and particle entrapment, amplifying both the humidity and dust degradation chains described in the sections above. This synergistic behavior implies that the substrate material properties controlling thermal resilience cannot be optimized in a vacuum: one must consider the moisture intake principles that govern humidity and dust vulnerability. For instance, polyimide offers superior thermal stability among flexible polymer substrates, but absorbs substantially more water by mass than other flexible polymers, potentially compromising the electronics it thermally protects via moisture ingress at the conductor [28,33,57,69,76,81]. Therefore, it is pertinent that device designers approach substrate selections as an optimization problem, where thermal, hygroscopic, and contamination mechanisms are assessed in tandem.

#### 3.3.2. Mitigation Strategies

Mitigation of the effects of thermal degradation in flexible devices has been pursued through both materials engineering and geometric design. Huang et al. demonstrated intrinsically temperature-resistant flexible strain sensors by pairing a positive temperature coefficient material with a negative temperature coefficient material, achieving a net thermal drift of only 160 ppm/°C by cancellation of thermal responses [40]. Li et al. took a different approach, exploiting the atomic configurational disorder of high-entropy alloy nanofibers to induce lattice distortion that suppresses electron–phonon scattering, reaching a TCR of 45.59 ppm/°C [19]. Within their design, Liu et al. demonstrated that a Peano fractal conductor geometry reduced thermally induced stress to 69.03% to its linear baseline, while the addition of multiwalled carbon nanotubes bridged microcracks during deformation and prevented its propagation into device-level failure [14,47]. For applications requiring survival at temperatures more extreme than the capability of any polymer substrate, Alhendi et al. demonstrated that printed electronics on alumina ceramic substrates with gold conductors operate reliably up to 850 °C; however, this sensor is merely to establish an upper bound to printed device thermal resilience and has no effective flexibility [43]. Polyimide, with a glass transition temperature of approximately 360 °C and a CTE of roughly 20 ppm/°C, remains the most capable polymer substrate for flexible electronics under thermal stressors [79]. However, as discussed in Section 3.2, its high moisture absorption of approximately 2% introduces a tradeoff between thermal and humidity resilience. A more comprehensive discussion of thermal mitigation strategies, including substrate selection and design approaches, is presented in Section 4.

#### 3.3.3. Limitations and Caveats

Despite growing interest in flexible electronics and its applications, significant gaps remain. Existing thermal cycling studies on flexible devices employed accelerated life testing with temperature extremes and cycling rates that may not accurately reflect the thermal variations encountered in deployment. In addition, field data remains scarce [24,35]. The mitigation strategies demonstrated in Section 3.3.2, although promising, have been validated only in strain sensor applications with (relatively) narrow temperature ranges and whose transferability to other types and applications of sensors has not been established. Moreover, no studies have addressed the synergistic substrate optimization problem explored in this review: simultaneously balancing thermal, hygroscopic, and particulate resilience in flexible sensors and electronics. The existing literature evaluates these properties in isolation—thermal studies seldom characterize moisture uptake or particulate contamination via microcracks—leaving device design without a unified material selection for deployment about multiple environmental stressors. Developing such a framework, in comparative testing of substrate materials across thermal, hygroscopic, and contamination stressors simultaneously, represents an essentially unaddressed field in flexible electronics.

## 4. Mitigation

Having established that all three degradation mechanisms reviewed in this study are governed by shared properties (Section 3), this section examines to what extent existing mitigation strategies address, or fail to address, those shared properties. The degradation mechanisms expressed in this review identified mitigation strategies in the context of its given environmental stressor (Section 3.1.2, Section 3.2.2 and Section 3.3.2). Examined independently, each strategy effectively addresses its stressor. By contrast, as expressed in Section 3.3.1, flexible electronics in deployment do not encounter environmental stressors in isolation. The central question for mitigation is therefore not whether individual strategies work for individual stressors, but whether they remain effective, compatible, and non-interfering when combined. This section seeks to organize the mitigation strategies addressed into four categories—encapsulation and barriers, substrate selection, conductor and sensor material engineering, and geometric design—and analyze where these categories converge, diverge, or conflict, and what research is needed to resolve the resulting tradeoffs.

### 4.1. Mitigation Strategies

#### 4.1.1. Encapsulation and Barriers

Encapsulation and barriers represent the most immediate cross-occurring mitigation strategy, as they operate by placing a low permeable layer between a device and its environment. This strategy inherently addresses both moisture absorption and particulate deposition. The PTFE filter demonstrated by Chen et al. exemplifies this dual function, where, although designed primarily for humidity filtration, the membrane’s structure simultaneously excludes particulate in a single barrier [56]. Conformal polymer encapsulation achieves a similar purpose. Zou et al. demonstrated that an Ecoflex™coating dramatically reduced humidity-induced degradation over a 30-day period while maintaining mechanical flexibility. This provides direct experimental evidence for conformal polymer encapsulation as a viable humidity mitigation strategy [38]. Kazani et al. demonstrated that a thermoplastic polyurethane (TPU) coating applied over textile antennas helped protect printed conductors and reduced conductor degradation through 20 cycles, whereas uncoated antennas exhibited cracking after only 10 cycles—evidence that conformal coatings can simultaneously protect against moisture, abrasions, and chemical exposure in wearable application [44,82]. Similarly, a hybrid inorganic–organic barrier such as the AL_2_O_3_ Parylene bilayer demonstrated by Xie et al. achieved moisture protection in implanted neural devices over multiple years [59]. Li et al. demonstrated flexible film in a large area (TFE) encapsulation on PET substrates that achieved water-vapor transmission rates as low as $1.65\times 10^{-4}$ g/m^2^/day [42]. Additionally, the TFE barrier shows a hydrophobic performance, sufficiently improving the lifetime of water-sensitive flexible electronics, suggesting that a single encapsulation layer could simultaneously address both moisture ingress and particulate deposition [42]. Lewis established that barrier requirements range from approximately $10^{-1}$ g/m^2^/day to below $10^{-6}$ g/m^2^/day for OLED encapsulation, providing a quantitative framework for the levels of protection needed in flexible sensor applications [75]. The convergence across these studies is evident: barrier-based strategies that block moisture penetration inherently prevent particulate contamination and, in some cases, provide mechanical protection against delamination and abrasion. For devices exposed to both humidity and dust contamination, encapsulation and barriers offer the most immediate mitigation by disrupting the electrochemical degradation at its first step: formation of an electrolyte.

The relationship between encapsulation strategies and thermal mitigation is less straightforward. Any conformal coating inherently imposes a thermally insulating layer between the device and its environment, which produces two opposing effects on the degradation pathways expressed in Section 3.3. On the one hand, thermal insulation dampens the amplitude of temperature fluctuations in the device, which, as discussed in Section 3.3, may actually reduce thermal cycling fatigue by slowing the expansion and contraction rates that drive the cracking of the CTE mismatch. However, the same insulating layer prevents the dissipation of heat under sustained thermal loading. However, it must be noted, neither effect has been quantified for encapsulated flexible devices, making the thermal impact of conformal coatings an inherently open-ended question. The PTFE membrane in Chen, L. et al.’s study reduced the sensor’s humidity response by 60.6%—desirable for a gas sensor that should be immune to humidity interference, but counterproductive for a humidity sensor whose function depends on moisture interaction with the sensing element [56]. The development of selectively permeable encapsulation and barrier materials could resolve the tradeoff between environmental resilience and sensing capability. The tradeoff is inherent to any barrier strategy: the same permeability reduction that protects a device from unwanted environmental exposure lessens its ability to interact with the environment it is designed to sense.

#### 4.1.2. Substrate Selection

Substrate material selection is the mitigation strategy with the broadest consequences, as the substrate simultaneously governs thermal stress, humidity degradation, and dust-induced electrochemical failure. The analyses in Section 3 demonstrated that no single polymer substrate is optimal for all three environmental stressors [32]. Polyimide offers the highest thermal stability, but its moisture absorption maintains the availability of water at the conductor interface and contributes to lower reliability in humidity tests compared to liquid crystal alternatives [28,33,57,79,81]. PET offers moderate resistance to moisture but is completely embrittled by prolonged exposure to humidity at 85 °C at 85% RH. FEP absorbs negligible amounts of moisture but exhibits oxidation and crystalline changes under thermal aging [28,71]. Yang et al. introduced aramid nanofiber and graphene oxide substrates that maintain structural integrity in remarkably wide thermal ranges, pointing to a pathway of substrates designed for thermal resilience from the ground up rather than being constrained by the inherent limitations of common polymers [83]. Choi et al. compiled a comprehensive comparison of glass transition temperatures and CTE between candidate flexible polymers, providing a broad framework for the optimization of substrate selection identified in this review as needed [76]. The implication of this tradeoff suggests that the optimal substrate for a flexible device cannot be determined by evaluating thermal, hygroscopic, or contamination properties independently but requires simultaneous assessment of all three.

In contrast to encapsulation and barrier strategies, substrate selection is largely stressor-specific. The mitigation strategies addressed in Section 3.3.2 both target the electron–phonon coupling mechanisms that produce temperature-resistant sensing materials. These approaches are fundamentally complementary to humidity and dust mitigation strategies: a device can employ high-entropy alloy nanofibers for thermal stability simultaneously with a PTFE membrane for environmental protection as they operate at different functional layers. Salem et al. demonstrated a useful example of moisture–temperature interaction under humid conditions. They showed that Joule heating during energization evaporates the contaminant wet layer. In this limited sense, thermal effects can mitigate humidity-induced conductive degradation, demonstrating how stressor degradation mechanisms interact and counteract [60]. The development of flexible composite substrates would address and mitigate the multistressor optimization problem expressed throughout the review; however, this approach remains scarce in the context of flexible electronic resilience.

#### 4.1.3. Geometric Design and Conductor Material Engineering

Geometric design strategies, notably the fractal conductors demonstrated in Liu et al., represent a mitigation approach that is (currently) validated only for thermal stress, but whose underlying principle may transfer to other stressor contexts. The Peano fractal geometry reduced the thermally induced stress to 69.03% of its baseline linear layout [14,47,84]. In flexible PCBs under water-drop testing, Noh et al. found that electrochemical migration accelerated as the distance between electrodes with opposite bias decreased, while the time to dendritic bridging increased as the electrode distance increased [29,31,63,84]. This result was demonstrated in the context of dust-induced electrochemical migration, hence supporting the broader principle that conductor geometry can influence environmental resistance by altering the failure path length. Hygroscopic substrates that absorb and release moisture inherently undergo expansion and contraction cycles compared to those produced by thermal variation, generating stress at the substrate conductor connection through swelling rather than thermal expansion. Whether fractal conductor geometries can mitigate hygroscopic cycling stress as effectively as they mitigate thermal cycling stress requires validation. Xiao et al. noted that nanocellulose-based humidity sensors maintained operational stability through more than 2500 bending cycles, suggesting that the mechanical durability of the sensor under repeated deformation is achievable through material choice as well [20]. Likewise, self-healing conductor materials, such as the autonomously recovering semiconducting polymer demonstrated by Oh et al., suggest a pathway in which degradation from thermal cracking or moisture-induced delamination could be intrinsically repaired or reversed [45]. Likewise, Sun et al. developed a conductive hydrogel with water-responsive contraction, repeatable behavior in water, and solvent-assisted recovery after dehydration for conformal physiological and motion monitoring [46].

#### 4.1.4. Synthesis

**Figure 4:**
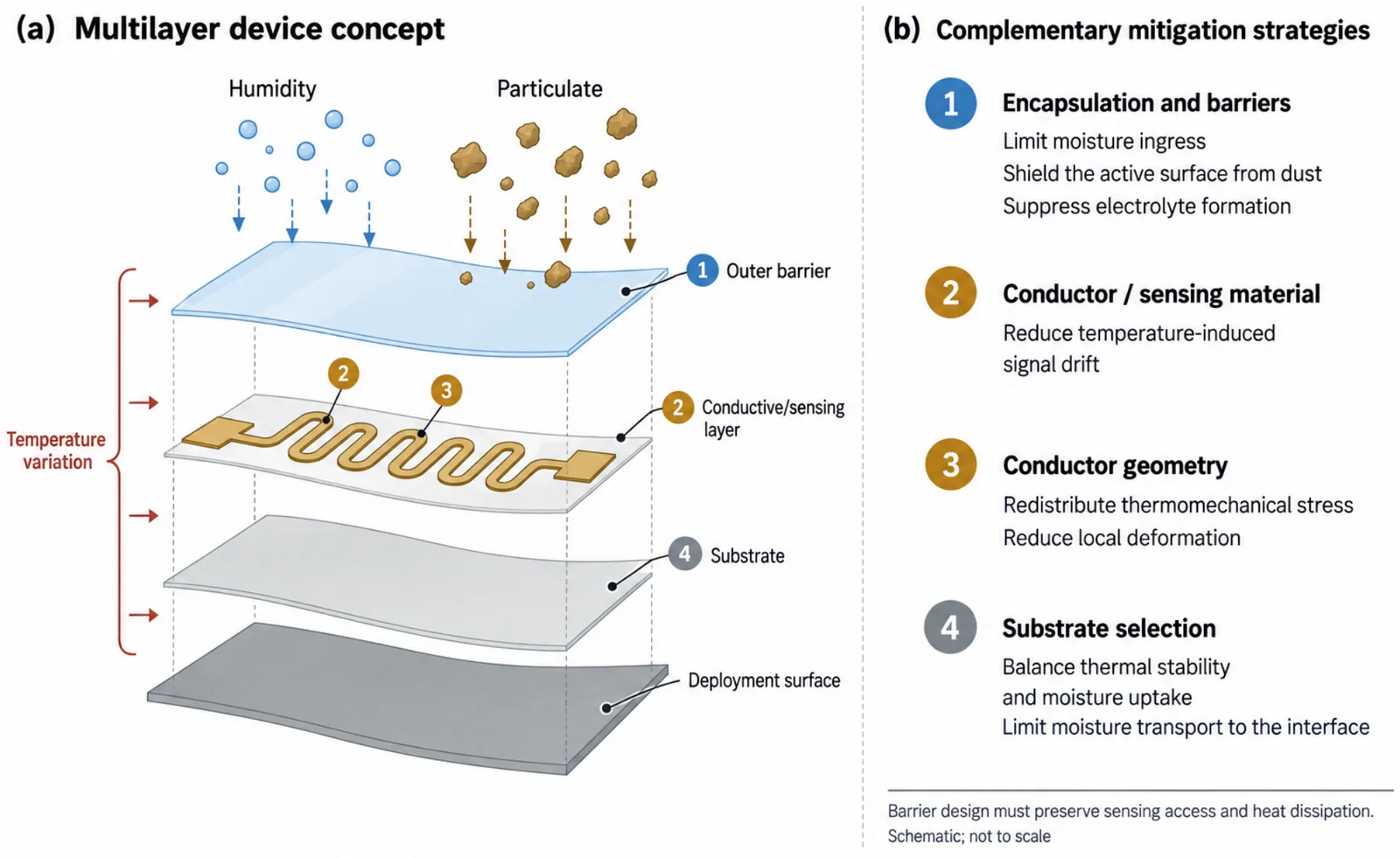
Multilayered architecture for mitigating environmental stressors, demonstrating design loci: (1) encapsulation and barriers; (2) conductor or sensing material; (3) conductor geometry; and (4) substrate selection. These loci are candidates to be qualified for a given deployment, not universally required layers.

Evaluating these four strategy categories together reveals natural convergent and divergent factors that suggest a layer mitigation architecture for environmental deployment. This layered architecture is illustrated in Figure 4.

In the outer layer, encapsulation and barrier strategies simultaneously address humidity and dust contamination, disrupting the electrochemical chain at its first steps. At the substrate layer, material selection governs the vulnerability of the device to all three stressors, with the material choice reflecting the dominant environmental stressor to the intended deployment of the device. At the conductor layer, the sensing material engineering compensates for thermal drift independently of the barrier and substrate strategies that precede it. At the geometric level, layout optimization distributes thermomechanical stress regardless of material choices in all other layers. This layered approach implies that multistressor-resilient devices are best achieved not through a single material or design solution, but through the combination of complementary strategies operating at differing functional scales [49,55].

Despite these converging factors, several unresolved divergences define research for multistressor mitigation strategies. The most consequential is the substrate optimization tradeoff described in Section 3.3: polyimide offers excellent thermal resilience but absorbs significant moisture; the moisture resistance of PET enables hydrolytic embrittlement, and the excellent resistance of FEP to moisture is converse to its inferior mechanical and thermal performance. This tradeoff cannot readily be resolved by selecting any one existing substrate, which requires either new substrate developments, high glass transition temperature, low CTE, and sufficient mechanical flexibility, or the development of a composite substrate that combines the appealing properties of multiple polymers. A second unresolved conflict is the sensitivity/protection tradeoff in environmental sensors: any encapsulation or barrier that blocks humidity and dust may also lessens the sensor’s interaction with the intended measurement. Developing selectively permeable barriers represents a material science challenge at the intersection of sensor design and environmental stressor mitigation. The PTFE membrane explored in Chen, L et al.’s work provides partial proof of concept, functioning as both a particulate barrier and gas sensor, but its simultaneous reduction in response sensitivity illustrates the caveat of this approach [56]. Additionally, whether conformal encapsulation imposes a thermal penalty remains sparse in the literature.

### 4.2. Prospective Design Framework

The mitigation strategies reviewed here demonstrate that no single material or design choice solves the multistressor optimization problem addressed within this review. Encapsulation strategies that effectively mitigate moisture and particulate ingress through deposition introduce thermal and sensing tradeoffs (Section 4.1.1). Substrate materials that excel thermally absorb the most moisture (Section 4.1.2). Geometric design strategies verified for thermal stress have not been extensively tested under deposition (Section 4.1.3). These tradeoffs cannot be universally resolved as the optimal mitigation approach for deployment is environmentally dependent. For example, a textile antenna deployed against human skin in a tropical climate faces a fundamentally different cofactor profile than an antenna in an arid desert, yet both devices are subject to the same underlying degradation mechanisms.

This section synthesizes the findings of Section 3 and Section 4 into a prospective framework that maps measurable deployment conditions to mitigation strategies across all four mitigation strategies. The framework is organized as a three-dimensional array by which each axis corresponds to one of the environmental stressors reviewed: levels of particulate contamination, relative humidity, and temperature. Each axis is defined by thresholds derived from the literature reviewed in Section 3, allowing a device designer to classify a target deployment and the corresponding mitigation architecture using known or measurable environmental parameters, resulting in 27 distinct design prescriptions by environment. To the authors’ knowledge, no comparable framework currently exists for flexible sensors and antennas.

However, the framework is not intended to produce 27 distinct prescriptions; instead, it maps the prospective sufficient mitigation architecture for each individual environmental classification. At high severity, the framework converges by design: deployments that sustain continuous ionic particulate contamination, sustained critical relative humidity, and extreme thermal cycling should engage most of the mitigation strategies, as each intends to address a distinct degradation pathway that other strategies (outside of synergistic properties) cannot cover. The convergence of the mitigation architecture at higher levels of the array therefore reflects a genuine cofactor coupling established in Section 3.

#### 4.2.1. Environmental Classification

**Figure 5:**
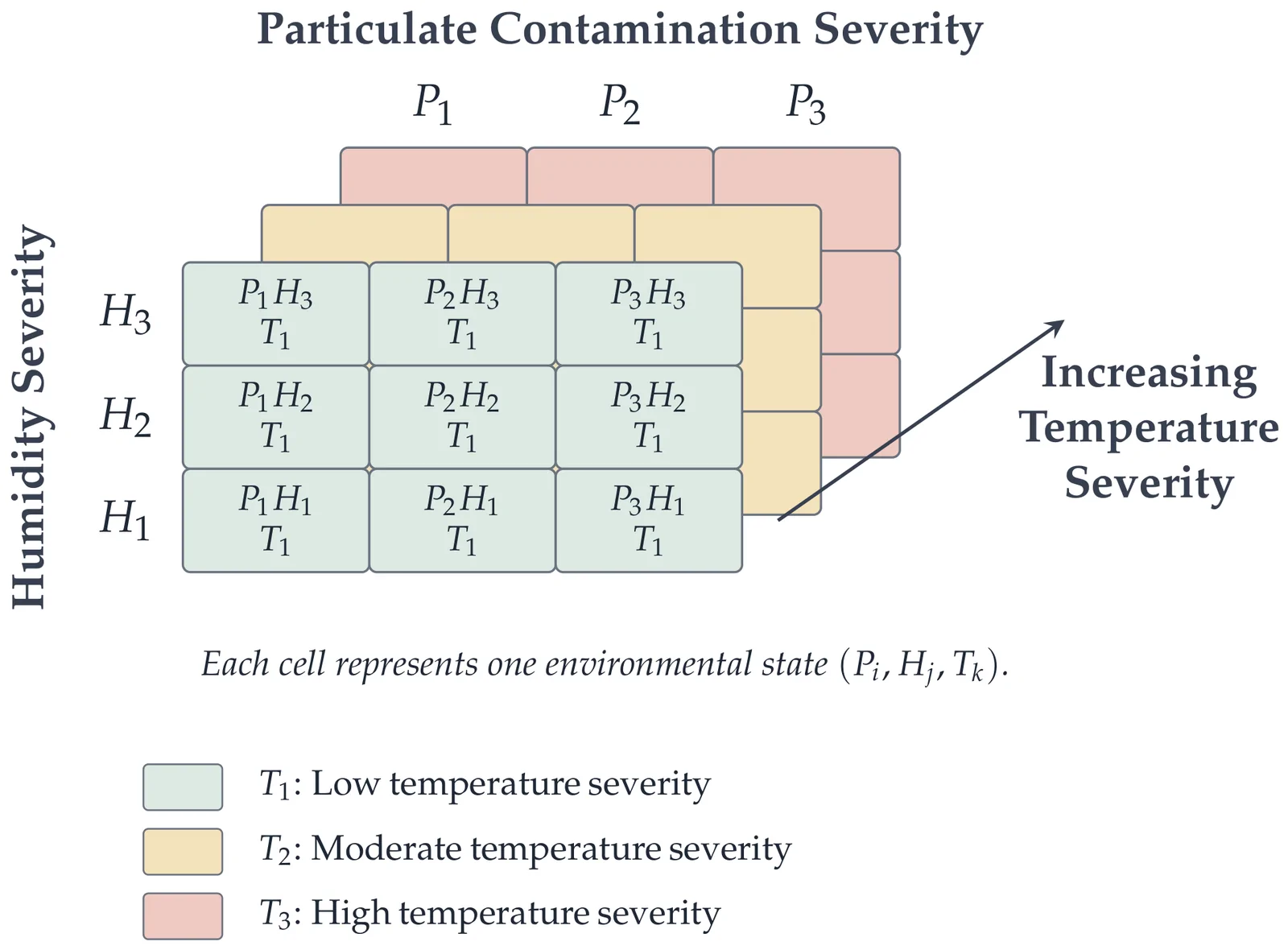
Structure of the provisional P–H–T array. Each cell corresponds to one environmental state. Every cell ($P_{i},H_{j},T_{k}$) carries candidate barrier, substrate, conductor, and geometric design considerations.

The framework classified deployment environments along three axes, currently divided into three severity levels defined by preexisting thresholds, as shown in Figure 5. The threshold values are selected at empirical boundaries where the respective degradation mechanisms undergo transitions as described in the literature reviewed in Section 3.

##### Axis #1: Particulate Contamination Severity

The severity of dust-induced degradation is not only governed by the mass of the deposit, but also by its ionic content and hygroscopic composition, which determine the conductivity of the electrolyte formed upon moisture ingress and thus the subsequent severity of the electrochemical degradation mechanisms demonstrated in Section 3.1 [23,50,51,61,63,65]. Song demonstrated that the aqueous ionic conductivity of particulate samples provides a clear indicator of the severity of subsequent electrochemical degradation, with indoor dust showing a mean conductivity of 2526 μS/cm compared to 1640 μS/cm for outdoor dust due to higher concentrations of ionic compounds through cleaning products, human excretion, and building materials [23,26,27]. Lu et al. corroborated this relationship, demonstrating that different ionic contents produced distinct failure times and pathways in solder alloys, affirming that electrolyte content rather than particulate mass governs the degradation rate [23,26,49,50,51,61].

This review covers three peer-reviewed publications that report tabulated conductivity values for real dust or aqueous ionic solutions derived from real dust: Wang et al. [85], Wang et al. [86], and Chrzan [87]. Of these, Wang et al. provided a characterized data set with values spanning across multiple sites and sample sizes [85]. The combined data define three levels of conductivity: from 12 μS/cm (PM_2.5_ minimum; Wang et al.) to 2210 μS/cm (aqueous CaSO_4_; Chrzan). In urban settings, the conductivity of aqueous dust is between 200 and 1000 μS/cm, with an average of 450 μS/cm [85,86,87]. For concentrations exceeding ≈2.5 mS/cm, the literature surrounding real dust extract is not well characterized; the high conductivity in this range is anchored by reference-grade aqueous electrolyte data rather than field extracts. Standard conductivity tables report aqueous salt conductivities well above this range, including CRC aqueous solution tables, Gilson conductivity tables, and industrial conductance compilations derived from experimental electrolyte data [88]. Note that these values should be interpreted as an upper bound, not a direct field measurement.

Three levels of severity of contamination are defined, with ionic conductivity of the aqueous dust solution as a first-order screening parameter, not as a complete predictor of particulate contamination severity (corrosion or electrochemical migration). Values are defined in Table 2. Additional factors include dust loading per unit area, particle size distribution, ionic species, chloride and sulfate concentration, pH, hygroscopicity, critical/deliquescence relative humidity, time of wetness, surface roughness, voltage, electrode spacing, and the nature of conductor materials.

Where aqueous ionic conductivity cannot be measured, deposited mass or surface coverage can practically, but not necessarily equivalently, indicate particulate contamination severity [27]. P remains unassigned until a dust sample is characterized.

##### Axis #2: Humidity Severity (Activation Cofactor)

The role of humidity in the degradation of flexible sensors and antennas is governed by its relationship to the critical relative humidity (CRH) of the ionic salts present in the deposited particulate. CRH defines the relative humidity (RH) threshold by which hygroscopic deposits absorb enough ambient moisture to form an electrolyte film, activating the electrochemical degradation chain expressed in Section 3.1. Song identified CRH values ranging from 40% for ammonium bisulfate to 79% for ammonium sulfate at room temperature, establishing that typical indoor humidity conditions (40% to 60% RH) may be sufficient to activate the electrochemical chain for most common salts [23,26]. Three RH levels are defined based on sustained ambient humidity relative to thresholds of common environmental particles [23], as summarized in Table 3.

##### Axis #3: Temperature Severity (Acceleration Cofactor)

Temperature variation degrades flexible electronics via three distinct pathways: electrical drift, mechanical deformation due to the coefficient of thermal expansion (CTE) mismatch between layers, and structural failure due to cumulative cyclic fatigue (Section 3.3). As the dominant degradation mode is qualitatively different between these mechanisms, the severity of the temperature is defined by (a) the sustained operating temperature range and (b) the cycling amplitude (the difference between the maximum and minimum temperature experienced). The resulting classification is summarized in Table 4.

#### 4.2.2. Decision Rules

Each of the four mitigation strategies suggested in Section 4 advances through differing levels of severity according to a defined trigger condition tied to the thresholds established above. The rules were applied across all 27 cells. Abbreviations are defined in Table 5.

##### Encapsulation and Barriers

The barrier strategy is upgraded according to the activation logic of the deposit-humidity-induced electrochemical degradation chain. E-min may be sufficient only when neither moisture nor ionic content can sustain a surface electrolyte (*P*1 ∩ *H*1), because RH < 40% lies near the CRH of even the most hygroscopic salts identified in Song et al. [23,26]. E-conf is utilized when particulate or humidity alone enables electrolyte formation: sustained RH ≥ 40% enables hygroscopic deposits to deliquesce, and aqueous conductivity κ≥150μS/cm may provide sufficient ionic conductivity for ECM once in the presence of device-specific sufficient RH [25,38,42,56]. E-seal may be considered when *both* conditions are met with great severity (P3 with H2 or H3, or H3 with P2 or P3), as at this point, the electrolyte forms rapidly and persistently and conformal coatings alone cannot provide the WVTR reduction required to significantly slow moisture ingress [25,38,42,56,59,75].

##### Substrate Selection

As substrate material determines mechanical properties across particle deposition, humidity, and temperature, substrate selection is driven by the dominant stressor or by stressor pairs that no single polymer can simultaneously mitigate. S-any is acceptable only at T1 with at most P2 or H2, where neither thermal nor moisture transport governs failure. S-lowWVTR may be considered when humidity is the dominant stressor at a low or moderate thermal severity, as the WVTR of the substrate governs how moisture permeates about the conductor interface, degrading the device [28,32,57]. S-PI is utilized in T2 or T3 with moderate humidity (H2 ≤ H), as the polyimide demonstrates exceptional thermal resilience [28,76,81]. The S-composite reflects the substrate optimization problem identified in Section 3.2.2 rather than any specific commercial material and corresponds to the principal experimental gap identified in Section 7. S-composite may be considered when both T ≥ T2 and H ≥ H2, as no single polymer satisfies thermal stability and low moisture absorption [28,32,83].

For a device-specific composite substrate, qualification may consider glass transition or softening behavior, CTE over the operating range, moisture uptake, WVTR, modulus, bending radius, adhesion, process compatibility, dielectric constant, and loss tangent at the operating frequency [28,58,89,90].

##### Conductor Material

C-std is acceptable when neither the ECM nor thermal drift is significant. C-resist could be considered when ECM is enabled (P ≥ *P*2 ∩ *H* ≥ *H*2), with material selection following the dendrite resistance ranking established for flexible PCBs by Noh et al. [29,50,62]. C-TCR may be considered at severe temperature, where thermal drift on printed antennas (≈3.5 dB change in *S*_11_ from 25 to 90 °C) [41]) becomes consequential. C-HEA may be considered when T3 conditions are coupled with elevated humidity or particulate, where conventional metallic films cannot mitigate thermal drift and corrosion resistance [40].

##### Conductor Geometry

G-std is acceptable when no stressor dominates. G-space may be considered when ECM is enabled (P ≥ P2 ∩ H ≥ H2), where the increased electrode space extends the migration path and delays dendritic bridging, as demonstrated by Noh et al. [23,26,29]. G-fractal may be considered at severe temperature (T ≥ T2), where thermomechanical stress concentrates at bends and initiates microcracks that can lead to the ingress of particulate and moisture [14,47,84]. When both conditions are met, the geometric layout must address both failure modes simultaneously, reflecting that fractal design and electrode spacing operate on independent modes and are not interchangeable.

These decision rules are summarized in Table 6.

#### 4.2.3. Design Suggestions by Environment

The three-axis classification in this review produces 27 unique environmental combinations, each representing a distinct cofactor profile for which a specific mitigation architecture is proposed. The design suggestions for each combination are organized across the four mitigation strategies established in Section 4, whose complete framework is presented in Table 7, Table 8 and Table 9. Each cell in the tables provides a brief mitigation architecture, in addition to an evidence classification: recommendations supported by direct experimental evidence on flexible or printed electronics are indicated in **bold**; recommendations extrapolated from the cofactor logic developed in Section 3, but lack direct experimental validation are indicated in *italics*.

##### Validation Priorities

Among the extrapolated recommendations, first, validation priority is assigned to those transferred from rigid PCB or MEMS studies. Second, validation priority is assigned to the remaining extrapolated recommendations. These are author-assigned priorities.

##### Illustrative Application Cases

- **Outdoor flexible RFID tag.** If site measurements establish P2/H3/T2, the framework suggests E-seal, S-composite, C-resist + C-TCR, G-fractal + G-space as candidate design considerations.
- **Body-worn patch.** Assign P only from characterized deposits and consider H/T from environmental and skin-side measurements.

## 5. Other Considerations

The environmental framework developed above addresses three stressors—particulate contamination, humidity, and temperature—that the literature identifies as the primary drivers of degradation in flexible sensors and antennas. However, deployed devices are subject to additional stressors that, while beyond the scope of this review, warrant acknowledgment. This section briefly addresses each and identifies synergistic effects with particulate contamination, humidity, and temperature.

### 5.1. Device-Specific Outcome Parameters

While the P–H–T framework guides exposure classification and design considerations, it does not establish a specific device performance or failure threshold. Considerable outcome parameters may include:

1. **Flexible sensors.** Sensitivity drift, baseline drift, hysteresis, response/recovery time, repeatability, calibration stability, selectivity, and signal-to-noise ratio.
2. **Flexible antennas and RFID systems.** Resonance frequency shift, $S_{11}$, impedance matching, bandwidth, gain, radiation efficiency, radiation pattern, polarization, read range, and backscatter performance.
3. **Flexible interconnects and PCBs.** Resistance change, surface insulation resistance, leakage current, dendritic bridging, and open/short failure.

### 5.2. Ultraviolet Radiation

Polymer substrates commonly used in flexible electronics are inherently susceptible to photodegradation after prolonged exposure to ultraviolet (UV) radiation [91,92,93,94]. The primary mechanism through which photodegradation occurs is through the photo-oxidative chain scission: UV photons are absorbed by chromophoric groups within the polymer backbone, breaking polymer chains, generating free radicals, and reducing the molecular weight, causing deterioration of the polymer’s mechanical properties—such as surface roughness or brittleness—and oxidation under exposure to air. Ultimately, this results in the degradation of the polymer over an unpredictable amount of time [91]. Specifically, Harito et al. demonstrated that the polyimide exhibited a significantly increased capacity to adsorb a Methylene Blue (MB) solution upon exposure to UV [94].

#### 5.2.1. Synergistic Effects

The relevance of UV degradation is two-fold. First, photo-oxidation chain scission reduces the mechanical integrity of the substrate; therefore, the fatigue threshold for CTE mismatch failure under thermal cycling is decreased, essentially shifting the device about the T axis (Section 4.2.3) without altering the thermal environment. Second, UV-induced surface roughening increases the available surface area for particulate deposition and moisture ingress, effectively increasing the effective deposit and relative humidity.

#### 5.2.2. Mitigation Strategies

Yousif and Haddad proposed five strategies for stabilizing polymers: (1) light screeners, (2) UV absorbers, (3) excited-state quenchers, (4) peroxide decomposers, and (5) free radical scavengers. Of these strategies, it is widely believed that excited-state quenchers, peroxide decomposers, and free radical scavengers are the most effective [91]. Similarly, Harito et al. demonstrated that, unlike neat PI, the titanate nanotube (TiNT/PI) compound exposed to prolonged UV demonstrates a much slower rise in the concentration of adsorbed MB. Hence, shorter cracks (≈40 μm long) appeared in TiNT/PI while (>100μm) were visible in neat PI under prolonged UV exposure [94].

### 5.3. Mechanical Deformation

Mechanical deformation—be it bending, stretching, twisting, or compression—constitutes a fundamental challenge for flexible electronics [95,96,97,98,99]. Unlike particulate contamination, humidity, and dust, which are ambient stressors acting on a stationary device, mechanical deformation is intrinsic to flexible devices and their applications: wearable sensors flex with movement, conformal antennas bend around nonuniform surfaces, and rollable devices undergo cyclic deformation. The dominant failure mechanism under cyclic deformation is conductor failure, whereby repeated strain accumulation induces microcracks at stress concentration [100,101,102] points—such as the conductor–substrate interface)—which culminate into either resistance loss or delamination, ultimately leading to device failure [96,99].

#### 5.3.1. Synergistic Effects

The interaction between mechanical deformation and the stressors reviewed in this paper is bidirectional. First, deformation-induced microcracks function near indistinguishably from the thermally induced CTE mismatch microcracks, creating entrapment sites for particulate deposition and humidity ingress. Second, environmental exposure degrades the fatigue resistance of the device. Humidity-induced swelling introduces residual stress that lowers the crack initiation threshold [103,104]. The literature has shown that copper conductors suffer fatigue under cyclic deformation, whereas silver nanowires, graphene, and carbon nanotubes offer superior strength but remain more susceptible to the primary environmental stressors and their degradation mechanisms reviewed in Section 3 [105,106,107,108,109].

#### 5.3.2. Mitigation Strategies

The geometric design strategies discussed in Section 4.1.3, notably the fractal conductor geometries demonstrated by Fan et al. and Liu et al., were originally intended for mechanical strain distribution as opposed to thermal mitigation [14,47]. Their prevalent inclusion in the thermal mitigation framework demonstrates the properties of mechanical deformation and the shared materials of thermal cycling, and perhaps the same geometric mitigation strategies. However, the interactions between particulate contamination and mechanical deformation on flexible substrates have not been widely experimentally investigated to the authors’ knowledge.

### 5.4. Additional Factors

Additional factors, such as chemical exposure, sweat contamination (in wearables), biofouling, and washing, can further influence degradation in deployed flexible sensors and antennas, although their implications are beyond the scope of this review [110,111,112,113]. Nevertheless, these additional factors can amplify the mechanisms described above. For example, as illustrated in Section 3.1, ionic sweat can increase surface conductivity under humid conditions, accelerating the electrochemical degradation chain through contamination [23,26,27,37]. Therefore, further work should evaluate these and other additional factors, in combination with the environmental framework developed in this review.

## 6. Limitations and Evidence Boundaries

Controlled particulate-deposition studies on flexible sensors and antennas remain scarce. Rigid electronics and flexible interconnect studies provide transferred mechanistic context [23,29,48]. Direct RFID thermal-cycling evidence also exists [24], but neither category validates complete particulate–humidity–temperature packages or establishes equivalent effect magnitudes, failure times, or mitigation performance across devices.

Exposure methods, contaminants, device conditions, and endpoints vary across studies (Section 3.1), so reported values are not directly equivalent [23,26,27,50,61]. Aqueous-extract conductivity is only a protocol-dependent indicator of soluble ionic contamination, not a complete electrochemical-risk predictor or a measure of dry-particle effects.

Likewise, the study-specific transfer limitations documented in Table 1 constrain generalization and evidence transferability to flexible devices under dust exposure, particularly where rigid device evidence differs in contaminant and exposure conditions.

The framework’s 27 cells organize exposure information, candidate mitigations, and validation priorities, not service-life predictions, failure probabilities, or sufficient architectures. Cells remain author-derived, even where a component has experimental support. Representative target-device validation is required before prescriptive use.

Finally, although ultraviolet radiation and mechanical deformation may amplify the degrading effects of particulate contamination, humidity, and temperature, they are outside the primary scope of this review, and are therefore not considered within the P–H–T framework.

## 7. Conclusions

This review examined the effects of particulate contamination, humidity, and temperature variation on flexible sensors and antennas, synthesizing evidence from PCB research, MEMS studies, and flexible device investigations to develop a comprehensive and integrated account of environmental degradation mechanisms and their interactions. The analysis reveals that the three stressors do not operate independently, but form an interconnected degradation system whereby each stressor amplifies or abates the effects of others through shared material properties and synergistic mechanisms.

The findings in the humidity and moisture contamination analysis (Section 3.2) show that the moisture intake of the substrate, through capillary wicking and hygroscopic absorption, was identified as the central variable to govern the magnitude of degradation on different substrates. Dust contamination analysis (Section 3.1) established that particulate-induced degradation occurs through a linked electrochemical chain—deposition, hygroscopic absorption, ionic dissolution, resistance loss, corrosion, migration, and failure—in which each step is modulated by the ionic content, pH, and hygroscopic contents of the particulate. Synergistically, this entire chain is humidity-dependent and, without sufficient moisture, even heavily ionic dusts remain inert. The convergence of these mechanisms constitutes a key finding: the same material properties that determines a substrate’s susceptibility to humidity-induced dielectric degradation may govern its ability to form dust-derived electrolytes and persistently cause the electrochemical chain described in Section 3.1.

Thermal degradation analysis (Section 3.3) introduces an additional dimension to this synergistic mechanism. Temperature variation degrades flexible devices through electrical drift, deformation from CTE mismatch between substrate and conductor layers, and structural failure from cyclic fatigue-induced cracking. These mechanisms may be governed by the substrate and material properties that are largely independent of the humidity–dust electrochemical chain mentioned above. Although, the synergistic coupling between thermal and hygrothermal stressors is substantial: elevated temperature can accelerate moisture diffusion and humidity-induced degradation, thermal cycling causes persistent microcracks that serve as strongholds for moisture ingress and particle entrapment. Counterintuitively, the positive relationship between elevated temperature and conductivity, where prolonged heat exposure can improve performance through annealing, can be reversed in the presence of humidity. The substrate material that governs thermal resilience does not necessarily align with those that minimize moisture intake, once again illustrating the multistressor optimization problem that remains poorly characterized in the literature.

The mitigation analysis (Section 4) demonstrated that individual strategies cannot be assumed to be effective in multistressor environments without experimental corroboration. Encapsulation and barrier strategies converge effectively across both humidity- and dust-induced mechanisms, but its thermal management can introduce tradeoffs: conforming coatings may dampen temperature fluctuations that drive cyclic fatigue while simultaneously preventing heat dissipation. This review proposed a multilayered approach to mitigation in which encapsulation addresses humidity and dust in the outermost layer, the substrate material largely governs the baseline vulnerability across all three stressors, and the conductor material and geometric design can distribute thermal dissipation and dampening in the sensing and trace layers. However, this architecture assumes that environmental resilience is best achieved through the combination of complementary strategies and that experimental corroboration remains absent. Section 4.2 translates this layered architecture into a prospective three-axis design framework. Deployments are classified along three independently measurable axes—aqueous ionic conductivity of deposited particulate, sustained relative humidity, and operating temperature range with cycling amplitude—each divided into three severity levels defined by empirical transitions in the reviewed literature. The 27 resulting environmental combinations are assigned to specific mitigation prescriptions in encapsulation, substrate, conductor, and geometry, with each recommendation classified as either directly supported by experimental evidence on flexible or printed electronics or extrapolated from the cofactor logic developed in Section 3. To the authors’ knowledge, no comparable framework currently exists for flexible sensors and antennas. The framework is intended both as a design aid and as a structured map of where empirical validation is most urgently needed: cells dominated by extrapolated recommendations identify the highest-priority experimental gaps.

### Research Gaps and Validation Priorities

This review identifies four research topics that, taken together, may substantially advance the literature on the environmental reliability of flexible sensors and antennas. First, direct studies on the dust-induced degradation mechanism in flexible electronics appear scarce. The conclusions reached about dust-induced degradation are derived from *rigid* PCB and MEMS research, and, while mechanistically and materially analogous, do not directly transfer to flexible devices. Although, the scarcity of the literature surrounding dust-induced degradation mechanisms on flexible sensors and antennas demonstrates an addressed literary gap [23,26,29]. Second, no study has systematically reviewed how substrate moisture ingress predicts the relative severity of humidity-induced degradation in multiple substrate classes. Third, the development of composite flexible substrates would directly address the multistressor substrate optimization tradeoff that persists in single substrate electronics [83]. Fourth, the development of selectively permeable encapsulation and barrier materials is currently in its infancy and would resolve the sensitivity-protection prospective tradeoff that currently limits barrier strategies for sensing applications [56].

The central contribution of this review is the reframing of environmental reliability for flexible sensors and antennas from a collection of independent stressor-related problems into an integrated, multistressor optimization problem governed by shared material properties. The existing literature evaluates particulate contamination, humidity, and thermal stressors in isolation, leaving the device design without a unified basis for material selection for environmental protection. Developing such a framework, grounded in comparative experimental testing of multiple substrates across thermal, hygrothermal, and contamination stressors simultaneously, represents an essential step toward flexible sensors and antennas that can perform reliably under the environmental exposure they are designed to endure.

Finally, addressing some of the key knowledge gaps highlighted in this work could enable the development of mechanically robust, environmentally resilient flexible systems for wearable technologies, while also facilitating the exploitation of dust-induced surface degradation as a functional transduction mechanism for sensing applications.

## Figures and Tables

**Table 1:** Key study evidence provenance record.

Key Study and Reference	Device Category	Substrate	Conductor/Functional Material	Contaminant and Deposition Protocol	Environmental Exposure Conditions	Electrical/Mechanical/RF Metric	Mitigation Approach	Major Result	Transfer Limitation
Noh et al. [29]	Flexible PCB	PI; 25 μm thick	Bare copper; electroless Ni; electroless Ni/immersion Au finishes	Distilled-water droplet bridging comb patterns	Water-drop testing at 3–18 volts DC; pH 6.5	Time to dendritic bridging; polarization behavior; corrosion rate	Surface finish selection; wider electrode spacing and lower bias	Dendrite growth ranked Cu > Ni > ENIG	Liquid-water ECM evidence
Bollström et al. [30]	Inkjet-printed Ag tracks on paper	Multilayer-coated fine paper; kaolin/latex barrier and topcoat; commercial-paper comparators	Ag nanoparticles; spin-coated P3HT; drop-cast EMIM-TFSI/PS-PEO-PS ion gel	Water vapor	23 °C; tracks: 25–90% RH, 24 h; transistor: 90% RH (1 h), dry glovebox (18 h), 90% RH (1 h, then 24 h); separate dimensional cycling included 100 °C/30 min drying	Track surface resistivity; dimensional change/roughness; transistor output/transfer curves and on/off ratio	Barrier coating; top-side encapsulation recommended, not tested	On/off ratio fell from 22 bare to 12 after 1 h at 90% RH, 7 after drying/rewetting; modulation lost after final 24 h at 90% RH	Humidity-only paper-electronics evidence—not dust validation; substrate barrier protects only from below
Andersson et al. [31]	Flexible PCB	HP Advanced Photo Paper and Canon PT-101 photo paper	Silverjet DGP-40LT-15C ink, approximately 30 nm Ag nanoparticles in triethylene glycol monoethyl ether; 30 μm active lines	Paper-coating constituents mobilized by ink solvent; canon paper also dip-coated in 0.01 or 0.1 M NaCl, dried, and then printed; DI-water control	22°C; sequential 30%, 60%, 80%, and 90% RH; HP and Canon tests lasted 600 and 300 min, respectively	Resistance sampled every 27 s	Geometry and coating chemistry-tuned humidity sensitivity	HP patterns initially near 900 Ω and 1.5 MΩ converged toward 50 Ω after exposure. NaCl made Canon patterns more sensitive	Intentionally exploits humidity-dependent resistance; it does not establish universal corrosion or deposited-dust degradation
Hertleer et al. [25]	Flexible antenna	Cotton; flax/cotton; 50/50 cotton/polyamide; PET fleece; flexible PU foam	Copper-plated woven nylon patch and ground plane	Water vapor	23 °C; 10–90% RH in 10% increments; ≥24 h per condition	Return loss; resonance frequency; simulation-fitted $\varepsilon_{r}$; humidity-dependent tanδ analysis deferred	Low-moisture-regain substrates recommended	Increasing RH lowered resonance frequency; PET fleece (0.95% regain) and PU foam (1.99%) showed smaller shifts	Humidity-only textile RF evidence, not dust; designs prioritized sharp resonance over full-band coverage
Lahokallio et al. [28]	Flexible PCB	Kapton HN PI (50 μm); FEP (127 μm); Mylar PET (125 μm)	-	Water-vapor aging; separate deionized-water immersion for uptake measurements; no dust deposition	85 °C/85% RH; 500–6000 h; room-condition interruptions every 500 h; separate immersion: 20 min–5 days	Elastic modulus; thermal expansion coefficient; glass transition/melting temperatures; water uptake; crystallinity/chemical structure	-	PI modulus stable through 6000 h; FEP crystallinity increased, with mechanical deterioration/oxidation at 6000 h; PET hydrolysis-induced embrittlement after 2000 h, as reported	Not dust or complete-device validation; PI material stability does not establish moisture-safe interfaces
Zardetto et al. [32]	Flexible electronics substrate/conductor characterization	PET, heat-stabilized PET/PEN (125 μm); ITO-coated PET/PEN (175/125 μm); glass, stainless steel and Ti foils	ITO (15 Ω/sq on primary polymer samples); spin-coated PEDOT:PSS (110±10 nm); FTO and metal-foil comparators	Acetone/ethanol: 10 min ultrasonic baths and 3 h soaks; xylene/toluene/chlorobenzene: 2 h immersion	30 min heating, room temperature–250 °C for plastics and up to 600 °C for comparators; tensile/compressive bending, radii 30–3 mm, 1–150 cycles or held curved; UV, 225 mW/cm^2^ for 150 min	Sheet resistance after heating and during/after bending; deformation radius; crack morphology; optical transmittance	PEDOT:PSS overcoat; heat-stabilized polymers; two-edge restraint during heating	Thermal curvature reached 1 m radius at 115 °C for PET versus 180 °C for HS-PET/HS-PEN; PEDOT:PSS shifted PET/ITO fixed-tensile degradation onset from r<14 to r<11 mm	Substrate/electrode evidence—not dust, controlled-humidity or complete sensor validation
Frisk et al. [33]	Flip-chip interconnects on flexible PCBs	LCP (50 μm); PI (25 μm); thin FR-4 (100 μm)	Ni/Au-plated Cu tracks; Cu or Au bumps; 40 μm ACFs containing Au-coated nickel or polymer particles	50±5 g/L NaCl in deionized water, pH 6.5–7.2; separate water-vapor exposure	85 °C/85% RH, 5000 h, interrupted every 500 h; salt spray: 35±2 °C, 3000 h; thermal cycling: −40 to 125 °C, 30 min/cycle, 10,000–20,000 cycles	Daisy-chain resistance; time to failure; failure percentage	Substrate selection using LCP	20% LCP, 55% PI, 50% FR-4; at 3000 h: 90%, 100%, 100%, respectively	Interconnection evidence—not dust, bending or sensor validation
Davila-Frias et al. [34]	Flexible hybrid electronics	Flexible polyamide; thickness unspecified	Silicon die; conductive traces and anisotropic conductive paste; composition unspecified	Water vapor	65 or 85 °C with 85 or 98% RH; 150 h	Daisy-chain resistance; failure criterion: 20% resistance increase; modeled time to failure and reliability	-	Model-estimated mean time to failure: 143.8 h at 85 °C/98% RH versus 913.1 h at 65 °C/85% RH	Not dust validation; reliability extrapolation depends on linear-damage and normal-lifetime assumptions
Lahokallio et al. [24]	Flexible RFID tag	PET film (50 μm)	Al antenna; Au-bumped chip; epoxy anisotropic conductive paste containing Ni particles	-	Tests 1/2/3/4: minimum −40 °C; maxima 85/125/125/125 °C; soak per extreme 5/5/14/15 min; transition 1/1/1/105 min; duration 2000/1000/500/1250 cycles; RH unspecified	Reader threshold power versus frequency; failure criterion: doubled threshold power at optimal frequency; cumulative failures and Weibull lifetime parameters	-	All tags failed by 2000/1000/500 cycles in tests 1/2/3; some survived 1250 cycles in test 4; fast profiles primarily caused Al-antenna cracking, while slow test 4 caused ACP-interconnection cracking	Thermal-cycling evidence—not dust/humidity validation; profiles can change failure mechanisms, limiting service-life extrapolation
Merilampi et al. [35]	Printed RFID tag	Plain copy paper; 105 μm, 80 g/m^2^	Polymer thick-film Ag ink in polyester resin; 60–65 wt% Ag	Water-vapor exposure; separate single 2 s water dip; no dust deposition	−40 °C/168 h; 125 or 150 °C/24 h; 90 °C/65% RH/500 h; −55 to 100 °C, 200 cycles, approximately 150 min/cycle, 60 min/extreme	Threshold power; backscattered signal power; calculated theoretical read range versus frequency	Initial antenna tuning to a slightly higher frequency recommended to offset expected detuning	After 90 °C/65% RH/500 h, 2 of 3 samples had theoretical read ranges <1 m over most tested frequencies; unstressed reference peak just under 6 m at 860 MHz	Antenna/substrate aging—not complete-tag reliability; n=3/test; no particulate-dust validation
Gupta et al. [36]	Printed, stretchable strain array	PDMS; approximately 0.1 mm	DuPont Intexar PE671 carbon ink; PE874 silver interconnects; thin PDMS encapsulation	Humidifier-generated water vapor supplied through a pipe; no dust deposition	Separate tests: 60/70/80% RH at nominal 30 °C; 31/41/48 °C at 60% RH; 0–1 mm displacement in 0.2 mm steps; 4–5 loading–unloading cycles/condition	Resistance change ΔR; normalized resistance–strain response; baseline resistance; hysteresis	Basic PDMS encapsulation present; improved encapsulation and temperature compensation recommended	RH 60–80% increased hysteresis from 27.92% to 37.8%; at 60% RH, 31–48 °C reduced ΔR from 4.80 to 1.77 kΩ	No dust testing; selected elements—not full-array validation; combined temperature–RH interactions untested
Sandeep et al. [37]	Flexible antenna	Jute fabric	Caswell copper-paint patch and ground; stated paint specification <1 Ω/sq at 1 mil thickness	Pickering artificial eccrine sweat, pH 4.5; 15 sprays, brief partial dip, or 24 h immersion	Temperature/RH unspecified; 50 sweat-exposure repetitions reported	$S_{11}$/resonance; axial ratio; gain/efficiency; radiation patterns; Tafel-derived corrosion rate	Jute substrate selection	After 24 h immersion, the 3.5 GHz band was rejected; 4.9/5.8 GHz operation retained, with gains of 8.4/8.6 dBi and efficiency of 79–87%	Liquid-sweat evidence—not particulate- or dry-dust validation; corrosion-rate measurements do not establish service lifetime
Zou et al. [38]	Flexible sensor	Self-supporting hydrogel enclosed in Ecoflex	PAM/SA/polydopamine/PANI conductive hydrogel; nickel–chromium wire electrodes; conductive-carbon-tape comparator	Indoor-air exposure; hydrogel water loss/uptake; no dust deposition	Approximately 25 °C/60% RH for one month	Relative resistance change; gauge factor; compression hysteresis/response lag; mass change	Complete Ecoflex encapsulation with adherent interface	Reported weight/resistance changes during month-long storage: 1.9%/7.7% encapsulated versus 32.9%/580.8% unencapsulated	Ambient-storage moisture-stability evidence—not dust validation; no controlled temperature/RH sweeps
Li G. et al. [39]	Flexible Si-NM n-MOSFETs	Kapton film (12.5 μm); PI stress buffer and PDMS adhesive (≈5 μm each)	Si-NM; Cr/Au electrodes; SiO_2_/Al_2_O_3_ gate dielectrics; poly-α-methylstyrene interlayer	-	MOSFET heating: up to 300 °C, approximately 3 min/condition; PAMS-free control to 350 °C; RH unspecified	Transfer/output curves; threshold voltage, mobility, on/off ratio and gate leakage; resistor resistance; fracture morphology	PI buffer reduces thermal expansion mismatch	MOSFET transfer response retained after 250 °C/3 min, but channel current undetectable after 300 °C/3 min; PAMS-free control retained response after 350 °C	Intentional thermal destruction—not dust/humidity or RF validation
Huang et al. [40]	Flexible sensor	TPU substrate; laminated TPU encapsulation using 100 μm film	Screen-printed TPU/MWCNT/few-layer graphene/EMIM ionic liquid composite; sputtered Au layer	-	Temperature characterization: 0–60 °C; tensile strain: 0–80%; 2000 cycles at 10% strain	Relative resistance change; gauge factor; TCR; hysteresis; response/recovery times; NFC readout distance	Positive-TCR Au counterbalances negative-TCR carbon/ionic composite	Reported TCR: $160\times 10^{-6}$ °C^−1^ over 0–60 °C; peak GF: 2415.76 at 40–60% strain	Thermal-compensation evidence—not dust or controlled-humidity validation
Li W. et al. [19]	Flexible sensor	PVDF membrane; outer PU protective film	FeCoNiMnZn/PVDF nanofibers; annealing-induced HEA metallization; Ag-paste terminal contacts	-	−10 to 70 °C, 10 °C steps over 70 min; 6000 cycles at 30% strain across 0/30/60 °C; additional 1200 h aging; RH unspecified	Relative resistance change; TCR; gauge factor; response/recovery times; fatigue and aging drift	Intrinsically low-TCR HEA network; PU protective laminate incorporated	TCR: 45.59 ppm K^−1^; GF: 1.12 at 19.2–50% strain; resistance drift reached 4.79% after 6000 cycles	Thermal stability evidence—not dust, controlled-humidity or RF validation
Bhattacharjee et al. [41]	Printed chipless antenna temperature sensor	PVC; thickness unspecified	Ag conductive paint (RS 186-3600) loop; PEDOT:PSS sensing bridge	-	25–90 °C; ambient RH unspecified; separate lateral/cross-bending: 5.7/11.0 cm radii; 15–16 weeks	Resistance; $S_{11}$ magnitude and resonance frequency; VSWR; induced current	Thin transparent coating and proper storage recommended	Over 25–90 °C, sensing-element resistance decreased approximately 70% and antenna $S_{11}$ magnitude changed approximately 3.5 dB; thermal resonance shifts showed no systematic trend	Deliberate temperature sensing—not dust validation; lateral bending affects resonance
Li C. et al. [42]	Flexible encapsulation/barrier-film coupons	PET (190±5 μm); Si, quartz, glass, ceramic, and Cu test substrates	pEGDA/Al_2_O_3_ multilayers; fluorinated p(EGDA-co-PFDA) top layer	Water vapor; 8 μL DI-water and 10 μL hexadecane droplets; no dust deposition	WVTR: 38 °C/90% RH; polymer-coated Si aging: 2 years at 23±2 °C/50±5% RH, then 85 °C/85% RH for 8 days	WVTR; water/oil contact angles; water sliding angle; optical transmittance	iCVD/ALD organic–inorganic barrier stack; fluorinated micro/nanocone surface for water repellency	Four-dyad WVTR: $1.65\times 10^{-4}$ g m^−2^ day^−1^; optimum water contact/sliding angles: 168.4°/1.1°; aged Si-surface contact angle: $164.6\pm 2.0^{\circ }$	Coupon-level evidence; no device electrical/RF or dust-removal validation
Alhendi et al. [43]	Printed interconnects	Rigid alumina, 99.8% purity; DLP-printed boards	Au thick-film ink with glass-frit additives	-	Au adhesion aging: 300, 500, and 750 °C for >100 h; adhesive aging up to 750 °C/50 h; transmission-line measurements: 50–750 °C in 50 °C increments; antenna: 30–850 °C; RH not reported	DC resistance; substrate deflection; Au pull-off force; adhesive shear strength; $S_{11}$/$S_{21}$; dielectric constant/loss tangent; VSWR	Au/alumina material selection; 850 °C firing for adhesion; substrate prebaking above 100 °C to remove moisture; Ceramabond 503-VFG assembly adhesive	Dielectric constant and loss increased with temperature, with stronger permittivity growth above 400 °C; antenna VSWR remained 1–2 at 30–40 GHz across tested temperatures up to 850 °C	Rigid-ceramic thermal evidence only; no flexible substrate, bending, or particulate-dust validation
Kazani et al. [44]	Flexible microstrip antenna	20% cotton/80% polyester; polyester	Screen-printed Ag inks: DuPont 5025 (epoxy); Electrodag PF 410	Water/reference-detergent laundering; no dust deposition	20 washing cycles at 30±3 °C (ISO 6330, program 8A); dried, then conditioned at 20 °C/65% RH/24 h; tested every 5 cycles	$|S_{11}|$ at 2–3 GHz; resonance/bandwidth; visible cracking/delamination	80 μm TPU coating; more durable, flexible electrical connections recommended	TPU protected ink and prevented delamination through 20 cycles; some uncoated antennas remained functional; connector loosening after 15 cycles caused major RF degradation	Dry-state laundering results; connector failures confound RF changes; n=1/configuration; no dust validation
Oh et al. [45]	Stretch-able sensor array	SEBS substrate (100 μm); PDMS dielectric (2 μm)	DPP-TVT-PDCA/PDMS-PDCA-Fe blend; Au electrodes/interconnects	Artificial sweat drop-cast onto passivated array; composition/volume not reported; no dust	15 h sweat test; separate 0–30% array-strain tests; film self-healing for 24 h at room temperature	On/off drain currents; field-effect mobility; gauge factor; interconnect resistance; film fracture strain	100 μm SEBS passivation; dynamic Fe(III)–PDCA bonds for semiconductor self-healing	Array retained transistor switching through 15 h of sweat exposure; reversible strain response up to 30%	Liquid-sweat evidence only; no dust validation
Sun et al. [46]	Flexible sensor	Hydrogel film (∼0.6 mm)	PEG/α-CD/PEO/PVA network; imidazolium tetrafluoroborate ionic liquid; glycerol	Intentional water/glycerol–water immersion or dripping; no dust	Humidity tests: 25 °C/60–95% RH; water-loss test: 20 °C/7 d; repair assessed over 20 min at 25 °C/25% RH	Resistance/GF; conductivity; skin-interface impedance/ECG; stress–strain; mass retention; contraction	Glycerol for water retention; glycerol/water immersion to repair dehydration	Conductivity: 0.86 S/m; negligible contraction below 65% RH; stress, strain, and conductivity remained near initial values over six dehydration–repair cycles	No dust validation
Liu et al. [47]	Flexible sensor	PI, 75 μm; ICP-treated surface	Screen-printed Ag and In_2_O_3_/MWCNT thermo-electrodes	Anhydrous-ethanol droplets for evaporative thermal shock; no dust deposition	Hot-junction holds approximately 73–242 °C; cold junction approximately 24.5 °C; bending radii 2–6 cm; approximately 75/110/150 °C; RH unspecified	TEMF; Seebeck coefficient; relative resistance change; temperature drift; repeatability/hysteresis; response time	270° Peano geometry, MWCNT crack bridging, and co-conformal printing on a 6 cm radius surface	The 270° design’s maximum stress was 69.03% of the linear design’s value	Temperature-sensing/force-tolerance evidence—not dust or controlled humidity
Song et al. [26]	Rigid PCB	FR-4 laminate; 1.57 mm thick	Copper foil electrodes	Natural indoor and outdoor dust; pipetted 99.9% isopropanol suspensions	40°C; 40%, 60%, 80%, and 90% RH	AC impedance; aqueous-extract conductivity; pH	-	Marked low-frequency impedance loss observed at 60% RH for indoor dust; 80% for outdoor dust; dust-extract conductivities: 2526 versus 1640 μS/cm, respectively	Rigid devices do not establish flexible device or RF failure thresholds
Song [23]	Rigid PCB	FR-4 laminate; 1.6 mm thick	Copper interdigitated electrodes	Massachusetts indoor/outdoor dust, Tianjin outdoor dust, and Arizona test dust; pipetted 99.9% isopropanol suspensions; nominal loadings 1-4 mg/in^2^	50-95% RH at 40°C; temperature spanning 20-60°C at 80-90% RH	Impedance spectroscopy; DC resistance; time to failure	-	Dust-specific humidity transitions caused substantial impedance loss; more loading lowered transition RH; higher ion concentration in dust had the lowest time to failure	Rigid devices do not establish flexible device or RF failure thresholds
Song et al. [27]	Rigid PCB	FR-4 laminate; 1.6 mm thick	Bare copper electrodes	Natural outdoor dust; pipetted 99.9% isopropanol suspensions; loadings of 150, 300, 450, and 600 μg/cm^2^	50-95% RH at 40°C; temperature spanning 20-60°C at 80-90% RH	AC impedance magnitude/phase; bulk resistance	-	Dust-specific humidity transitions caused substantial impedance loss	Rigid devices do not establish flexible device or RF failure thresholds
Zhou et al. [48]	Rigid PCB	FR-4 copper-clad laminate	Copper traces with immersion-silver surface finish	Aqueous indoor dust; NaCl/Na_2_SO_4_ comparators; 2 μL droplets pipetted; concentrations 196-8439 mg/L	Water-drop testing; 3 volts DC	Surface insulation resistance; time to failure; dendrite growth and morphology	-	Concentrated solutions caused initial ionic conduction failure before ECM	FR-4 liquid-drop evidence, not dry dust or humidity onset
Lu et al. [49]	Solder–electrode ECM coupons	Ceramic	Sn-58Bi	5 μL NaCl or Na_2_SO_4_ droplets simulating salts	Water-drop testing; 3 volt bias	Mean ECM short-circuit time; current	-	Mean ECM failure time: 53 s in NaCl versus 32 s in Na_2_SO_4_; dendritic short circuits in both solutions	Ceramic-supported salt-droplet evidence—not natural dust-deposition or flexible device validation
Wang et al. [50]	Rigid PCB	FR-4; 0.8 mm thick	Bare copper	Beijing haze particles characterized; NH_4_Cl/(NH_4_)_2_SO_4_-loaded activated carbon; ethanol pipetted at 5 μL/cm^2^ and vacuum-dried	40 °C; 60% RH; 1, 8, and 24 h	Surface potential and corrosion morphology	-	Hygroscopic salts promoted surface electrolyte formation and localized corrosion	Rigid-PCB evidence—not flexible device/RF validation
Jellesen et al. [51]	PCB assemblies	Unspecified; nylon switch housing	Ag-plated brass terminals; Ag-clad bronze dome; Sn-plated steel legs	Flux residues; laboratory 1:20 flux/DI-water droplets	Liquid-drop ECM; 3 volts DC. Separate residue aging: 95% RH, 25–65 °C cycling	Leakage current–time; polarization curves; ECM bridging time	Minimize hand-solder flux; modify underlying copper layout to enable reflow/wave soldering; recommended, not tested	Failures were attributed to either insulating flux residues or corrosion/ECM associated with residues	Flux evidence—not controlled dust or flexible device validation
Yi et al. [52]	Rigid MEMS sensor	Silicon	Heater and four thermistors; material unspecified	Quartz sand; fan-assisted settling onto moist sensor, or, as reported, suspension spin-coating and drying	Approximately 20 °C, 60% RH; 0–19.1 m/s	Output voltage versus wind speed; simulated thermistor temperature difference	-	First-method output reductions were 5% and 24%; second-method average reductions were 1.91% and 3.75%, respectively	Quartz-sand surrogate; no flexible device/RF validation
Chen J et al. [53]	MEMS flow-sensor thermistor structure	Silicon; SiO_2_/SiN/SiO_2_ suspended beam	Pt thermistor (0.18 μm); SiN cover (0.8 μm)	Sand/dust (<149 μm); chamber deposition at reported 6 g/m^2^/day; 5 min blowing/5 min settling	Methods describe 2 h exposure of 12 samples with humidifier on/off; actual RH not quantified	Measured thermistor resistance/drift; baseline 270±30 Ω; simulated electrostatic current	Humidification attenuated resistance-reading disturbance	Maximum resistance decrease of 2.5 Ω unhumidified versus 1.3 Ω humidified; subsequent recovery; attributed to electrostatic measurement interference	No calibrated flow-error or flexible/RF validation; particle-size dependence simulated, not experimentally verified
deSouza et al. [54]	PurpleAir PA-II optical PM_2.5_ sensors	Not reported	Material compositions unspecified	Ambient particulate exposure	U.S. indoor/outdoor deployment, January 2017–July 2021; variable temperature/RH across climate zones; exposure was observational, not chamber-controlled	PM_2.5_ channel disagreement/flag rate; corrected concentration bias; permanent-degradation classification	Data flagging and RH-based correction; maintenance/replacement recommended, not experimentally tested	240/11,932 sensors (approximately 2%) classified as permanently degraded; corrected PM_2.5_ bias changed −0.12 μg/m^3^/year	No demonstrated dust causality or flexible/RF validation; higher PM exposure did not significantly modify normalized-error aging
Piotrowska et al. [55]	PCB SIR comb coupons	Comb substrate unspecified; separate FR-4/solder-mask specimens	Lead-free Sn100C; HASL finish	Palmitic, suberic, adipic, succinic, glutaric, and DL-malic acids; 5% wt/v IPA solutions pipetted and evaporated; approximately 100 μg/cm^2^	25, 40, 60 °C; 30–99–30% RH, 12 h per ramp; 50–99% RH, 1 h equilibration, 0–10 volt sweep at 2 mV/s	AC impedance; DC leakage; water uptake; ECM morphology	Flux-chemistry selection and enclosure climate limits recommended; no protective treatment tested	DL-malic impedance-derived deliquescence RH decreased from 75.4% at 25 °C to 59.4% at 60 °C; glutaric/DL-malic produced severe corrosion; higher temperature lowered the humidity threshold for water-layer formation	Not natural dust, complete flux formulations, or flexible/RF validation
Chen et al. [56]	MEMS sensor	Materials unspecified; epoxy upper cover	Metal-oxide sensing element; PTFE filter	Humid air from breath simulator introduced into sealed test box	34 °C; 75, 85, 95% RH; 20 L/min; 25 s. HAST: 130 °C, 85% RH, 96 h	Humidity-induced resistance change; retardation rate; alcohol response/sensitivity before and after HAST	PTFE membrane co-molded into the EMC cover	Humidity-response reduction, on average: 60.6, 59.9, 57.3% at 75, 85, 95% RH, respectively; all 25 specimens passed the stated HAST test	Not controlled dust filtration or flexible device validation
Watanabe et al. [57]	Polymer film characterization	Kapton (PMDA–ODA), UPILEX-S (BPDA–PDA); PET: Lumirror; all 125 μm	-	Pure-water immersion of prewashed/dried films; up to 420 min	Room-temperature immersion; DC measurements at 9–21% RH; capacitance measurements at 25 °C	Volume resistivity/conductivity (100 V DC); relative dielectric constant (1 V AC, 200 kHz–2 MHz, as stated in methods); water uptake	-	Equilibrium uptake: 2.7/2.5/0.5% for PMDA–ODA/BPDA–PDA/PET; PI conductivity increased sharply at water volume fraction ≈0.034; no corresponding PET transition	Material-level liquid-water evidence—not dust exposure or complete flexible-sensor/antenna validation
Pérez-Campos et al. [58]	Textile material characterization	Chopped cotton, polyester and polyamide	-	Water immersion to 400% mass gain; repeated 80 °C/2 h drying followed by cooling; no dust deposition	Moisture/density measurements: 25 °C; separate temperature sweeps: approximately 20–82°C	Dielectric constant $\varepsilon_{r}^{\prime }$ and loss factor $\varepsilon_{r}^{\prime \prime }$; bi-reentrant resonant cavity; 2.015–2.542 GHz	-	Moisture was the strongest influence; cotton dielectric constant/loss factor increased approximately 259%/564% as dry-basis moisture increased from 0 to 100%, as reported	Chopped bulk-fabric evidence—not intact antenna or dust validation; dry-basis moisture content is not ambient RH
Xie et al. [59]	Utah neural electrode arrays	Rigid silicon	IrO_2_ tips; Au wiring; Au–1% Pd inductive coil; Au/Sn flip-chip joints	Immersion in 1× PBS: 140 mM NaCl, 2.7 mM KCl, 10 mM phosphate	57 °C accelerated soaking; assumed aging factor Q=4 relative to 37 °C	Tip impedance; RF startup frequency/signal strength; active-array current	52 nm ALD Al_2_O_3_ + 6 μm Parylene C bilayer	At 37 °C-equivalent times: stable RF near 910 MHz/−50 dBm (hand receiver) through 1044 days; current ∼3 mA through 228 days; median tip impedance 60 → 160 kΩ by 960 days	Rigid arrays—no bending or dust validation
Salem et al. [60]	33 kV rated polymeric insulators	Silicone-rubber housing over fibre-reinforced plastic core	Steel end fittings	Flow-on kaolin/NaCl coating; dried >7 h	Fog: 1.5 L/h; rain: 3, 6, 9 L/h; chamber T/RH not specified	Leakage current/surface conductance; FEM electric-field/current-density distributions	-	Leakage current increased during wetting and decreased after wetting stopped; at 9 L/h rain, modeled local fields reached approximately 3.6 kV/cm (type I) and 4.5 kV/cm (type II)	Bulk high-voltage insulation; no flexible device/RF validation

**Table 2:** Aqueous ionic conductivity classification levels for particulate contamination.

Level	Label	Aqueous Ionic Conductivity, κ	Physical Meaning
**P1**	Low	κ<150μS/cm	Captures the lower bound of the Wang et al. PM_2.5_ extract distribution [85]. Ionic content may be insufficient to drive meaningful ECM at any RH. Corresponds to rural or remote sites.
**P2**	Moderate	150≤κ<1200μS/cm	Spans the central mass of real urban dust extracts, such as means reported by Wang et al. of 433–470 μS/cm [85]. Ionic content may be sufficient to sustain ECM when RH exceeds CRH. Encompasses most temperate urban, suburban, and light-industrial outdoor environments.
**P3**	High	κ≥1200μS/cm	Above the upper end of the Wang et al. urban dust distribution, with a reported maximum of 1223μS/cm [85]. Includes Song’s outdoor dust at 1640μS/cm, gypsum saturation at 2210μS/cm, Song’s indoor dust at 2526μS/cm, and concentrated or NaCl-rich environments. Corresponds to heavy industrial, coastal, indoor cleaning-product residue, volcanic, and body-worn scenarios [23,26].

**Table 3:** Humidity severity classification levels for environmental degradation.

Level	Label	Sustained Relative Humidity	Physical Meaning
**H1**	Low	RH<40%	Below the CRH of ammonium bisulfate, the lowest among common environmental salts identified by Song [23]. Even highly hygroscopic particulates are unlikely to form a persistent surface electrolyte, leaving the electrochemical degradation chain largely inactive regardless of contamination severity. Substrate moisture uptake is also negligible, with polyimide absorbing less than 0.5% by mass [57]. Corresponds to arid desert climates, high-altitude deployments, and tightly controlled HVAC environments.
**H2**	Moderate	40%≤RH≤75%	Spans most of the CRH range reported by Song for common environmental salts (40% for ammonium bisulfate to 79% for ammonium sulfate) [23,26]. The most hygroscopic salts deliquesce within this window while higher-CRH salts such as ammonium sulfate remain inert, partially activating the electrochemical degradation chain with severity strongly dependent on the ionic composition of the deposited particulate. Corresponds to most temperate climates and standard indoor environments without dedicated humidity control.
**H3**	High	RH>75%	Approaches and exceeds the upper end of Song’s reported CRH range (≈79% for ammonium sulfate) [23,26], allowing essentially the full range of common ionic constituents to form conductive surface electrolytes and strongly activating the electrochemical degradation chain regardless of specific dust composition. Substrate moisture uptake reaches severe levels: polyimide absorbs ≈2% water by mass at 85% RH, PET undergoes progressive hydrolytic embrittlement, and FEP exhibits oxidation and crystallinity changes under prolonged combined thermal–humid exposure [28]. Corresponds to tropical climates, coastal regions, and body-proximal microclimates where perspiration can generate RH exceeding 90% near the skin surface.

**Table 4:** Temperature severity classification levels for environmental degradation.

Level	Label	Operating Extremes	Cycling Amplitude, ΔT	Physical Meaning
**T1**	Low	−10 to ${50\;}^{\circ }C$	$\Delta T<{30\;}^{\circ }C$	Well below the glass transition temperatures of polyimide (≈360 C∘) and PET (≈80 C∘) [76,79]. Electrical drift from temperature coefficient of resistance (TCR) is the dominant degradation mechanism, with reported values for metallic films and nanowires used in flexible sensing typically near 0.002–0.003 C−1∘ [78]. CTE-mismatch microcrack initiation has been documented on flexible devices only at substantially larger cycling amplitudes [24,47]. Corresponds to indoor, climate-controlled, and stable body-worn deployments.
**T2**	Moderate	−40 to ${85\;}^{\circ }C$	${30\;}^{\circ }C\leq \Delta T<{80\;}^{\circ }C$	The ${85\;}^{\circ }C$ upper bound coincides with the standard accelerated aging condition used by Lahokallio et al. [28]. CTE mismatch generates appreciable shear stress at substrate–conductor interfaces; thermal cycling in this regime induces microcrack initiation in conductors and adhesives [24,47], which then serve as entrapment sites for particulate and pathways for moisture ingress, coupling thermal cycling to the mechanisms in Section 3.1 and Section 3.2. PET, with $T_{g}\approx {80\;}^{\circ }C$ and CTE ≈20–30 $\mathrm{ppm}{/}^{\circ }C$ [76,79], approaches its glass transition at the upper bound. Corresponds to outdoor diurnal cycling, automotive cabin environments, and industrial monitoring near moderate heat sources.
**T3**	High	Beyond −40 to ${125\;}^{\circ }C$	$\Delta T\geq {80\;}^{\circ }C$	Exceeds the glass transition temperature of PET [76], effectively excluding it as a substrate. Cumulative cyclic fatigue produces irreversible cracking at CTE-mismatch zones, as demonstrated by Lahokallio et al. [24] for −40 to ${125\;}^{\circ }C$ cycling on RFID tags. Among polymer options, polyimide grades and composite materials may be considered, but their viability depends on specified temperature extremes, cycling range, and adhesion [28,32,76]. Temperature-shifted CRH [55] may activate electrochemical degradation at lower ambient humidity than expected. Corresponds to engine-proximal aerospace and automotive deployments, cryogenic–ambient cycling, and industrial process monitoring.

**Table 5:** Notation key for candidate mitigation recommendations.

Category	Notation	Meaning
Encapsulation	E-min	Minimal encapsulation; standard environmental protection only.
	E-conf	Conformal coating to reduce moisture exposure and particulate deposition.
	E-seal	Sealed encapsulation to block moisture ingress and particulate contamination.
	E-PTFE	PTFE-based breathable or hydrophobic barrier for moisture/contaminant exclusion while maintaining flexibility.
Substrate	S-any	No special substrate requirement; standard flexible substrate acceptable.
	S-lowWVTR	Low-water-vapor transmission-rate substrate to reduce moisture transport.
	S-PI	Polyimide substrate recommended for thermal stability and mechanical durability.
	S-composite	Composite or multilayer substrate requiring device-specific qualification for thermal, moisture, mechanical, and electrical requirements.
Conductor	C-std	Standard conductor material acceptable.
	C-resist	Corrosion-resistant conductor recommended.
	C-TCR	Low-temperature coefficient-of-resistance conductor recommended to reduce thermal drift.
	C-HEA	High-entropy alloy or advanced conductor recommended for severe coupled stress environments.
Geometry	G-std	Standard conductor geometry acceptable.
	G-space	Increased inter-electrode spacing recommended to delay dendritic bridging and reduce ECM risk.
	G-fractal	Fractal or strain-tolerant conductor geometry recommended to reduce thermomechanical stress concentration.

**Table 6:** Tier decision rules for environmental degradation mitigation.

Tier Upgrade	Trigger Condition	Mechanism It Addresses
E-min → E-conf	RH≥40% or κ≥150μS/cm	Provisional activation condition for ECM is met through either hygroscopic electrolyte formation or device-specific sufficient ionic conductivity [23,25,26,38,42,56].
E-conf → E-seal	(*P* ≥ *P*2 ∩ *H*3) or (*P*3 ∩ *H* ≥ *H*2)	Provisional activation conditions are met at high severity, requiring stronger moisture-ingress control than conformal coating alone can provide [25,38,42,56,59,75].
S-any → S-lowWVTR	*H* ≥ *H*3 with *T* < *T*2	Substrate WVTR governs moisture permeation near the conductor interface under humidity-dominated exposure [28,32,57].
S-any → S-PI	*T* ≥ *T*2 with *H* < *H*3	Glass transition, CTE mismatch, and thermal resilience govern substrate survival under elevated temperature [28,76,81].
S-PI → S-composite	*T* ≥ *T*2 ∩ *H* ≥ *H*2	A composite substrate may address competing requirements [28,32,83].
C-std → C-resist	*P* ≥ *P*2 ∩ *H* ≥ *H*2	ECM corrosion is enabled, requiring conductor materials with greater dendrite and corrosion resistance [29,50,62].
C-std → C-TCR	*T* ≥ *T*2	Thermal drift exceeds sensing or antenna tolerance, requiring conductor selection based on temperature coefficient of resistance [41].
C-TCR → C-HEA	*T*3 ∩ (*P* ≥ *P*2 or *H* ≥ *H*2)	Coupled thermal–environmental loading requires simultaneous resistance to thermal drift and corrosion instability [40].
G-std → G-space	*P* ≥ *P*2 ∩ *H* ≥ *H*2	ECM path-length sensitivity dominates; increased spacing delays dendritic bridging [23,26,29].
G-std → G-fractal	*T* ≥ *T*2	CTE-mismatch stress concentrates at bends and initiates microcracks that couple to particulate and moisture ingress [14,47,84].

**Table 7:** Mitigation recommendations at temperature level T1 (low).

	H1: Low Humidity	H2: Moderate Humidity	H3: High Humidity
	RH < 40%	40% ≤ RH ≤ 75%	RH > 75%
**P1: Low Dust** κ<150μS/cm	E-min/S-any/C-std/G-std	**E-conf** [25,38,42,56]/S-any/C-std/G-std	**E-conf** [25,38,42,56]/**S-lowWVTR** [28,32,57]/C-std/G-std
**P2: Moderate Dust** 150≤κ<1200μS/cm	*E-conf* [25,38,42,56]/S-any/C-std/*G-std*	**E-conf** [25,38,42,56]/S-any/C-resist [29,50,62]/**G-space** [23,26,29]	**E-seal** [25,38,42,56,59,75]/**S-lowWVTR** [28,32,57]/**C-resist** [29,50,62]/**G-space** [23,26,29]
**P3: High Dust** κ≥1200μS/cm	*E-conf* [25,38,42,56]/S-any/C-std/G-std	**E-seal** [25,38,42,56,59,75]/S-any/**C-resist** [29,50,62]/**G-space** [23,26,29]	**E-seal** [25,38,42,56,59,75]/**S-lowWVTR** [28,32,57]/**C-resist** [29,50,62]/**G-space** [23,26,29]

*Note.* Bold entries indicate provisionally supported recommendations; italicized entries indicate extrapolated recommendations. Abbreviations are defined in Table 5.

**Table 8:** Mitigation recommendations at temperature level T2.

	H1: Low Humidity	H2: Moderate Humidity	H3: High Humidity
	RH < 40%	40% ≤ RH ≤ 75%	RH > 75%
**P1: Low Dust** κ<150μS/cm	**E-min**/**S-PI** [76,79,81]/**C-TCR** [40]/**G-fractal** [14,47,84]	**E-conf** [25,38,42,56]/*S-composite* [28,32,83]/**C-TCR** [40]/**G-fractal** [14,47,84]	**E-conf** [25,38,42,56]/*S-composite* [28,32,83]/**C-TCR** [40]/**G-fractal** [14,47,84]
**P2: Moderate Dust** 150≤κ<1200μS/cm	**E-conf** [25,38,42,56]/**S-PI** [28,76,81]/**C-TCR** [40]/**G-fractal** [14,47,84]	**E-conf** [25,38,42,56]/*S-composite* [28,32,83]/*C-resist + C-TCR* [19,29,40,50,55,62]/*G-fractal + G-space* [14,23,26,29,47,84]	**E-seal** [25,38,42,56,59,75]/*S-composite* [28,32,83]/*C-resist + C-TCR* [19,29,40,50,55,62]/*G-fractal + G-space* [14,23,26,29,47,84]
**P3: High Dust** κ≥1200μS/cm	**E-conf** [25,38,42,56]/**S-PI** [28,76,81]/**C-TCR** [40]/**G-fractal** [14,47,84]	**E-seal** [25,38,42,56,59,75]/*S-composite* [28,32,83]/*C-resist + C-TCR* [19,29,40,50,55,62]/*G-fractal + G-space* [14,23,26,29,47,84]	**E-seal** [25,38,42,56,59,75]/*S-composite* [28,32,83]/*C-resist + C-TCR* [19,29,40,50,55,62]/*G-fractal + G-space* [14,23,26,29,47,84]

*Note.* Bold entries indicate provisionally supported recommendations; italicized entries indicate extrapolated recommendations. Abbreviations are defined in Table 5.

**Table 9:** Mitigation recommendations at temperature-level T3.

	H1: Low Humidity	H2: Moderate Humidity	H3: High Humidity
	RH < 40%	40% ≤ RH ≤ 75%	RH > 75%
**P1: Low Dust** κ<150μS/cm	**E-min**/**S-PI** [28,76,81]/**C-TCR** [40]/**G-fractal** [14,47,84]	**E-conf** [25,38,42,56]/*S-composite* [28,32,83]/*C-TCR + C-HEA* [19,40]/**G-fractal** [14,47,84]	**E-conf** [25,38,42,56]/*S-composite* [28,32,83]/*C-TCR + C-HEA* [19,40]/**G-fractal** [14,47,84]
**P2: Moderate Dust** 150≤κ<1200μS/cm	**E-conf** [25,38,42,56]/**S-PI** [28,76,81]/*C-TCR + C-HEA* [19,40]/**G-fractal** [14,47,84]	**E-conf** [25,38,42,56]/*S-composite* [28,32,83]/*C-resist + C-TCR + C-HEA* [19,29,40,50,55,62]/*G-fractal + G-space* [14,23,26,29,47,84]	**E-seal** [25,38,42,56,59,75]/*S-composite* [28,32,83]/*C-resist + C-TCR + C-HEA* [19,29,40,50,55,62]/*G-fractal + G-space* [14,23,26,29,47,84]
**P3: High Dust** κ≥1200μS/cm	**E-conf** [25,38,42,56]/**S-PI** [28,76,81]/*C-TCR + C-HEA* [19,40]/**G-fractal** [14,47,84]	**E-seal** [25,38,42,56,59,75]/*S-composite* [28,32,83]/*C-resist + C-TCR + C-HEA* [19,29,40,50,55,62]/*G-fractal + G-space* [14,23,26,29,47,84]	**E-seal** [25,38,42,56,59,75]/*S-composite* [28,32,83]/*C-resist + C-TCR + C-HEA* [19,29,40,50,55,62]/*G-fractal + G-space* [14,23,26,29,47,84]

*Note.* Bold entries indicate provisionally supported recommendations; italicized entries indicate extrapolated recommendations. Abbreviations are defined in Table 5.

## Data Availability

No new data were created or analyzed in this study. Data sharing is not applicable to this article.

## Abbreviations

ECM	electrochemical migration
CRH	critical relative humidity
DRH	deliquescence relative humidity
CTE	coefficient of thermal expansion
TCR	temperature coefficient of resistance
PI	polyimide
PET	polyethylene terephthalate
FEP	fluorinated ethylene propylene
PEN	polyethylene naphthalate
PVDF	polyvinylidene fluoride
RFID	radio-frequency identification
UHF	ultrahigh frequency
MEMS	microelectromechanical systems
PTFE	polytetrafluoroethylene
HEA	high-entropy alloy
RH	relative humidity
UV	ultraviolet
SIR	surface insulation resistance
WVTR	water-vapor transmission rate
TPU	thermoplastic polyurethane
TFE	thin-film encapsulation
ANF	aramid nanofiber
PCB	printed circuit board
RF	radio-frequency
SNR	signal-to-noise ratio
